# On-Chip Tuning of Superconductivity in Fullerides via Current-Driven Rb^+^ Intercalation

**DOI:** 10.1021/acsnano.6c02466

**Published:** 2026-06-09

**Authors:** Konstantin P. Shchukin, Oliver N. Gallego Lacey, Baptiste Coquinot, Jacek Jakowski, Jingsong Huang, Patrik Staudenmayer, Yannic Falke, Ram Prakash Pandeya, Alexander Grüneis

**Affiliations:** † Institut für Festkörperelektronik, Technische Universität Wien, Gußhausstraße 25, Vienna 1040, Austria; ‡ II. Physikalisches Institut, Universität zu Köln, Zülpicher Strasse 77, Cologne 50937, Germany; § CEA, Universite Grenoble Alpes, IRIG-Pheliqs, Grenoble 38000, France; ∥ 148492 Institute of Science and Technology Austria (ISTA), Am Campus 1, Klosterneuburg 3400, Austria; ⊥ Computational Sciences & Engineering Division, 6146 Oak Ridge National Laboratory, Oak Ridge, Tennessee 37831, United States; # Center for Nanophase Materials Sciences, Oak Ridge National Laboratory, Oak Ridge, Tennessee 37831, United States

**Keywords:** fulleride, intercalation, alkali metal, superconductivity, Raman

## Abstract

An in-operando electro-intercalation method for the on-chip synthesis of alkali-metal-intercalated materials and their Raman spectroscopic and transport characterization in ultrahigh vacuum (UHV) is developed. We apply this method to synthesize fulleride superconductors via Rb^+^ intercalation into a C_60_ film. During the intercalation, we monitor the stoichiometry via UHV-Raman spectroscopy and probe superconductivity via transport measurements. An increase of the superconducting transition temperature from 7.0 K to 14.5 K is observed when the stoichiometry is tuned from Rb_2.7_C_60_ to Rb_3_C_60_. In our experiment, an ionic Rb^+^ flux into the host material is induced by an applied electronic current via a Butler–Volmer-type mechanism. Electro-intercalation captivates through improved stoichiometric precision, the ability to smoothly vary stoichiometry via duration of current application, and the absence of a lower limit of the volume of the host material. It represents a powerful concept for the on-chip synthesis of intercalated materials, battery research, and beyond.

## Graphical abstract

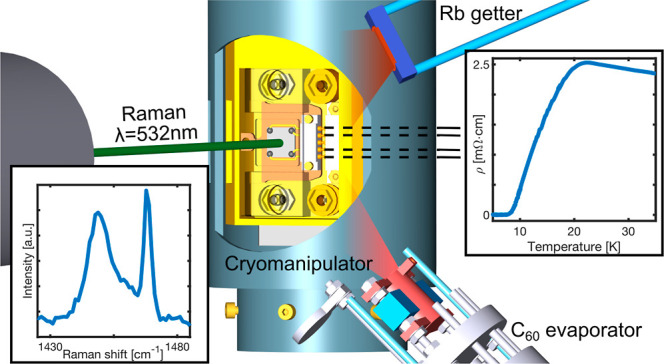

## Introduction

Ionic transport is of fundamental interest in materials science and is applied in chemical functionalization of fullerenes^1^ and van der Waals (vdW) materials,^2,3^ iontronics,^4,5^ and battery technology.^6,7^ Alkali metal ion intercalation can turn fullerenes into superconductors with an A_3_C_60_ stoichiometry (A = Rb, K, Cs). There is a renewed interest in superconducting fullerides for several reasons—their superconducting properties can be controlled by light,^8^ C_60_ grows epitaxially on topological insulators^9^ enabling superconductor-topological insulator interfaces, and the phase diagram of ternary fullerides is still poorly explored. Alkali metal intercalation into fullerenes has been achieved by intercalation from the vapor phase,^1,10–14^ liquid phase,^15,16^ and solid-state electrolytes.^17^ Vapor-phase intercalation of alkali metal ions requires an annealing step in order to disperse the alkali metal ions throughout the sample where they occupy tetrahedral and octahedral sites in between C_60_ molecules. For example, a temperature of ∼200 °C is required for synthesizing the bulk Rb_3_C_60_ phase^13,14,18^ which is superconducting around 30 K.^1^ Fulleride films have been studied via the van der Pauw method which was applied for vapor-phase-synthesized superconducting K_3_C_60_ on Si^19^ and for K_3_C_60_ and Rb_3_C_60_ films on the glass substrate.^20^ The traditional intercalation methods have limitations and impede progress in the field toward smaller films and more precise stoichiometries. Vapor- and liquid-phase intercalations have poor control over stoichiometry, and alkali metal intercalation via a solid-state electrolyte often leads to the degradation of the electrolyte.^21^ Especially, for small and thin fullerene films, tuning of the precise A_3_C_60_ stoichiometry by vapor-phase intercalation is a challenge. The required annealing step is not always possible on-chip, and the intercalation from the liquid phase cannot be performed in a device configuration. Fulleride superconductivity has been explained by the large density of states from the triply degenerate lowest unoccupied molecular orbital (LUMO) combined with strong electron–phonon coupling (EPC)^22–24^ to the H_g_(2) phonon around 440 cm^–1^.^25^ In addition, contributions to superconductivity from Jahn–Teller effects and electronic coupling mechanisms have been noted.^26,27^ Recently, an in-plane covalently bonded fullerene monolayer has been synthesized via Mg intercalation.^28^ In order to address the challenges related to stoichiometrically precise synthesis of fullerides and their in-operando characterization, a new experimental approach is required, which unites intercalation, spectroscopy, and transport measurements in a single setup.

In the present work, we perform on-chip synthesis of superconducting Rb_3_C_60_ using electro-intercalation of Rb^+^ into a fullerene film. We monitor the intercalation of air-sensitive Rb^+^ on-chip via Raman spectroscopy and electrical transport measurements in UHV conditions.^29,30^ Electro-intercalation is related to a current-driven overpotential across C_60_ grains that lowers the barrier for electron injection into the C_60_ grain. The injected electron reduces the energy required to detach and intercalate Rb^+^. The resulting kinetics are well captured by a Butler–Volmer description. By the applied direct current, we fine-tune the Rb stoichiometry *x* in-operando and verify *x* via UHV-Raman spectroscopy. For a given *x*, we then measure the temperature-dependent resistivity ρ­(*T*) and the superconducting transition. The fingerprint A_g_(2) Raman mode has an energy of 1469 cm^–1^ in bulk C_60_ and 1449 cm^–1^ in bulk Rb_3_C_60_,^25^ allowing determination of the stoichiometry Rb_*x*_C_60_. We measure the intensity of C_60_ and Rb_3_C_60_ modes during application of an electronic current to prove the Rb^+^ intercalation.

## Results and Discussion

### In-Operando Rb Intercalation and Sample Characterization

Figure 1a depicts the sketch of the experimental setup^30^ with the sample plate with four contacts mounted on a cryomanipulator, an inverted flange with a long working distance optical objective (NA = 0.55), the Rb getter source (SAES), and the C_60_ evaporator inside a UHV chamber. The cryomanipulator is mounted on a stage with encoded motors so that Raman mapping can be performed in UHV. Figure 1b shows the substrates that have been used in this study: (1) Al_2_O_3_ wafers and (2) silicon wafers with a 20 nm thick Al_2_O_3_ film grown by Atomic Layer Deposition (ALD) as shown in Figure 1b (Ia and Ib). Using UHV-compatible conductive silver epoxy, the substrate corners were contacted via 20 μm Au wires to the spring-loaded contact pins. Upon insertion of the sample plate into the cryomanipulator, all pins are electrically connected to the nanovoltmeter and precision current source outside UHV. This setup allows us to transfer the sample out from the cryomanipulator into a heater stage for in situ annealing (not shown). After loading the electrically contacted sample plate into the cryomanipulator, we performed evaporation of 12 monolayers (MLs) of C_60_ onto the sapphire (Figure 1b II) followed by Rb evaporation (Figure 1b III). This entire process is monitored real-time by UHV-Raman spectroscopy and resistivity measurements enabling precise doping control. Figure 1c shows a photograph of the Omicron-type sample plate with four spring-loaded pin contacts attached to the sample in van der Pauw geometry.^31^ The backside of the sample is attached to the sample holder using a droplet of the same UHV-compatible conductive silver epoxy. This floating configuration prevents any electrical shortening of the sample surface toward the holder during or after Rb deposition. In order to determine the location of the laser spot on the sample, we measured the distances relative to the four contacts which we identified by collecting the reflected light in a camera (Figure S1).

**Figure 1:**
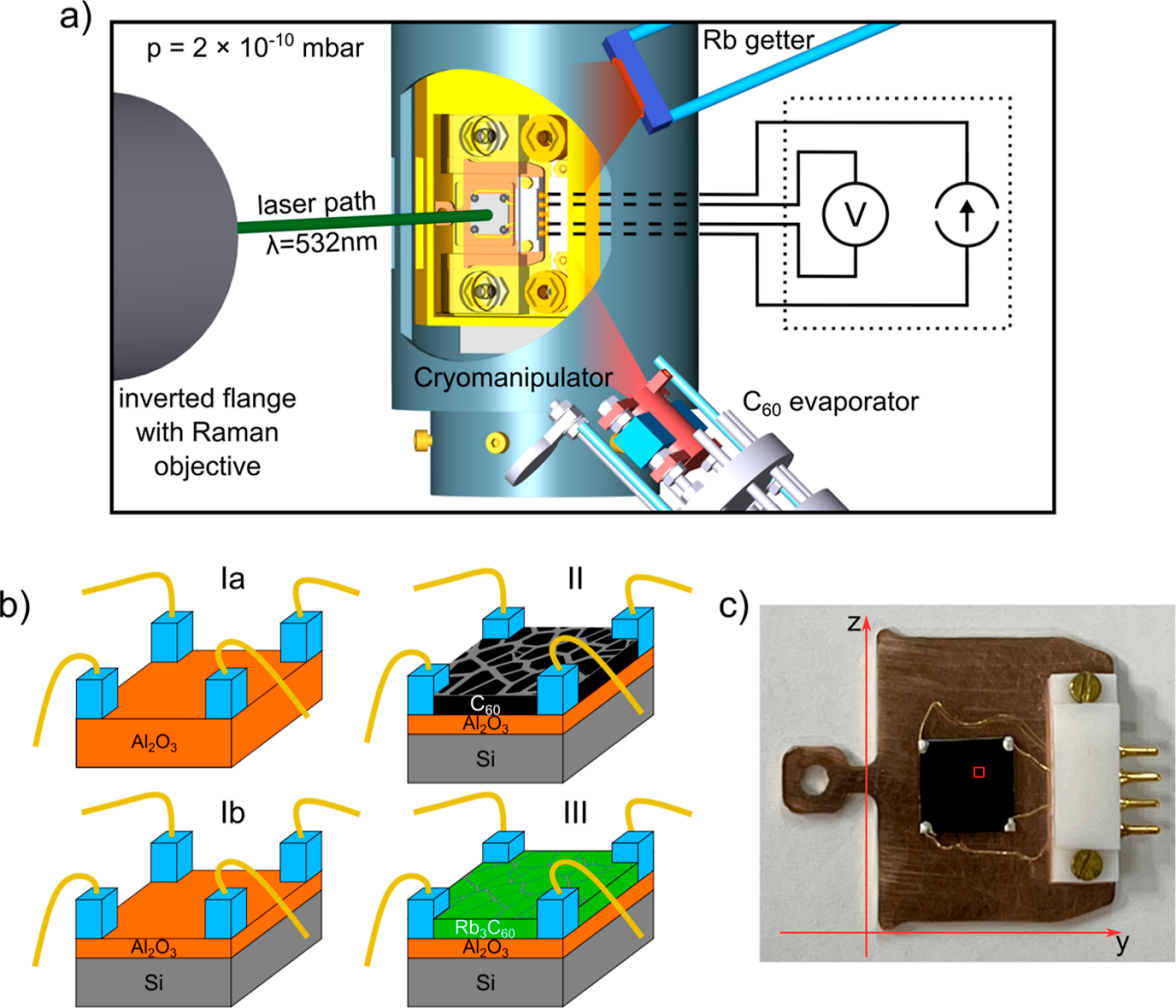
(a) Sketch of the setup for in-operando synthesis of Rb_*x*_C_60_. (b) Sample contacts and growth of Rb_3_C_60_. Ia: sapphire wafer with four contacts. Ib: 20 nm ALD-grown Al_2_O_3_ on Si. II: C_60_ on Al_2_O_3_/Si. III: Rb_3_C_60_ on Al_2_O_3_/Si. (c) Photograph of a sample holder with the contacted sample and the four spring contacts. The red square indicates the scanning window of 0.5 mm × 0.5 mm where the Raman map in Figure 2 was recorded.

The UHV-Raman spectrum of the C_60_ film on sapphire is shown in Figure 2a along with a typical spectrum of a Rb-doped film with Rb_2.85_C_60_ stoichiometry in the range 1430–1470 cm^–1^ which corresponds to the most intense A_g_(2) phonon mode. This sample was synthesized via evaporation of Rb onto the C_60_ film in UHV at 300 K. The UHV-Raman spectra were recorded at *T* = 5 K. This temperature was independently verified by measuring the superconducting transition of a Nb film.^30^ Due to its large Raman cross section, the spectra of several MLs of doped and undoped C_60_ can be efficiently measured with a large signal/noise ratio. The Raman spectrum of the alkali metal-doped fullerene molecule depends on its charge state and has distinct frequencies for the observed phases: neutral, *x* = 1, *x* = 3, and *x* = 6.^25,32^ For a neutral phase, it is a single peak at 1470 cm^–1^, Rb_1_C_60_ has a peak at 1458 cm^–1^, Rb_3_C_60_ at 1449 cm^–1^, and Rb_6_C_60_ at 1432 cm^–1^. For a mixed phase, several of these peaks appear simultaneously. After determining the relative Raman cross sections for each phase, we used Raman spectroscopy to determine the stoichiometry *x* of a sample with an arbitrary stoichiometry and Raman components from C_60_, Rb_1_C_60_, and Rb_3_C_60_ (see Methods and Figure S2). This procedure was used for the determination of *x* of the sample shown in Figure 2a and yields *x* = 2.85. Doping homogeneity is confirmed by Raman mapping of the A_g_(2) peaks inside the energy window shown in Figure 2a (see Figure S3 for individual scans). The small red square in Figure 1c indicates the extent of the spatial map. Figure 2b shows a Raman map of the stoichiometry *x* in Rb_*x*_C_60_ from a Raman line shape analysis yielding a spatial average of the stoichiometry of *x* = 2.85. Figure 2c shows Raman maps of the peak areas from the line shape analysis for the A_g_(2) peak of the initial pristine C_60_, the A_g_(2) peak corresponding to Rb_3_C_60_, and the peak of pure C_60_ that remains after evaporation of Rb (indicated as remaining C_60_). The Raman maps demonstrate high lateral homogeneity of both pristine and doped films, with relative standard deviations of the fitted peak areas in a range between 1.3 and 4.8%.

**Figure 2:**
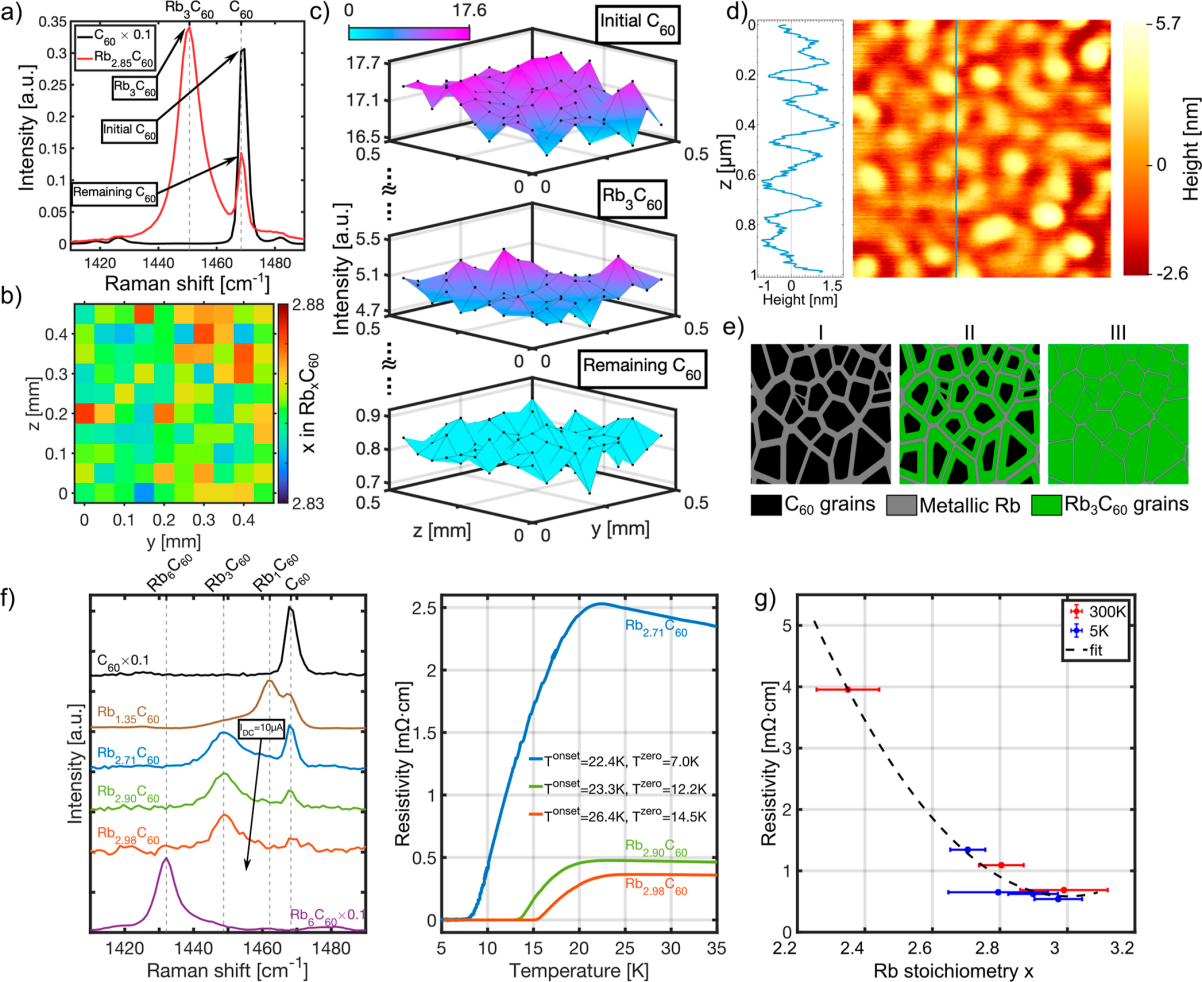
(a) Integrated Raman spectrum of Rb_2.85_C_60_ from the maps shown in (b) and (c). (b) Raman map of the stoichiometry *x* and (c) corresponding Raman maps of the initial A_g_(2) C_60_ peak and two A_g_(2) peaks of Rb_3_C_60_ and remaining C_60_. (d) Atomic force microscopy image of a 15 nm thick C_60_ film on Al_2_O_3_ scanned in window 1 μm × 1 μm. (e) Sketches of C_60_ grains (I) and partially (II) and fully (III) intercalated C_60_ grains with small amounts of Rb left in between the grains. (f) Raman spectra of Rb_*x*_C_60_ and resistivity ρ characterization. 10 μA current was used to intercalate Rb. (g) ρ versus stoichiometry at *T* = 300 K. All Raman and transport measurements were carried out in ultrahigh vacuum.

The sample morphology of our C_60_ films was probed by atomic force microscopy (AFM). A typical AFM image of the C_60_ film on a sapphire is shown in Figure 2d. The AFM confirms the nanostructured nature of the film reported in previous measurements^19,33^ of K_3_C_60_ and AFM measurements of C_60_.^34^ AFM analysis reveals that the 15 nm thick film consists of C_60_ grains with a lateral correlation length of 49 nm and an average roughness of 0.693 nm. The results of the AFM analysis are used in the modeling of the Rb^+^ intercalation described later. The nanostructured nature is crucial for Rb intercalation because it provides a large effective surface,^35^ according to which the Rb intercalation is progressing from the outside of the grain toward the inside. Hence, a partially intercalated C_60_ grain has a C_60_ core and an outer Rb_*x*_C_60_ shell separated by a moving intercalation front. The progressive Rb intercalation into C_60_ grains is shown in Figure 2e. The excess Rb that is not intercalated into a grain forms a network of conducting channels that run in between C_60_ grains. Because of the relatively poor electrical conductivity of the network of Rb channels, a significant voltage drop can appear across the film. This situation is reminiscent of a metallic wire network across which large voltage drops have been observed.^36^ The voltage drop across the sample is important for the intercalation mechanism, as described later.

We now discuss Raman and electrical transport measurements of in situ synthesized Rb_*x*_C_60_. Figure 2f depicts UHV-Raman spectra for sample stoichiometries between undoped (*x* = 0) and fully doped (*x* = 6) fullerides. For stoichiometries in the range *x* = 3, fullerides were metallic, and ρ­(*T*) was recorded in addition to Raman. For metallic fullerides, we can determine *x* from Raman and correlate it with an ρ­(*T*) trace and the appearance of superconductivity. In Figure 2f, we synthesized the phases Rb_1.35_C_60_ and Rb_2.71_C_60_ conventionally by two consecutive steps of Rb evaporation and annealing. In order to precisely control the stoichiometry for *x* beyond *x* = 2.71, we performed electro-intercalation of Rb^+^ by passing a 10 μA current through the sample for ∼96 h at *T* = 300 K which allowed for precise control of the stoichiometry. The electro-intercalation experiment was performed with the identical setup used for the van der Pauw measurements. For electro-intercalation, the applied current was ten times larger than for resistance measurements. In order to supply the Rb, electro-intercalation was performed during the deposition of ∼170 MLs of Rb. We also performed electro-intercalation without Rb evaporation onto the C_60_ film. In this case, an excess amount of Rb was made available by deposition prior to the electro-intercalation. This deposited Rb acts as a reservoir from which Rb for electro-intercalation was supplied (see the next section). The electro-intercalation shown in Figure 2f was stopped at *x* = 2.90 and at *x* = 2.98 after which the sample was cooled down to *T* = 5 K. Hereafter, a Raman spectrum and a ρ­(*T*) trace (right panel of Figure 2f) were recorded. The Raman fits of stoichiometries of Figure 2f are shown in the Supporting Information (Figure S4). Both the Raman spectra and ρ­(*T*) confirm Rb^+^ intercalation and the increase in stoichiometry *x*. Our experiments reveal that electro-intercalation at room temperature is a slow process with a rate of stoichiometry change Δ*x* = 3 × 10^–4^ per 1 h. The rate of stoichiometry change corresponds to an advance of the intercalation front by a distance of 0.83 pm per hour. Hence, stoichiometry tuning is enabled by this method with a high precision that cannot be achieved by other intercalation methods. The ρ­(*T*) for the three stoichiometries (*x* = 2.71, 2.90, and 2.98) showed vanishing resistance at low temperatures which is consistent with previous reports.^19,33^ This indicates that samples prepared by electro-intercalation have consistent superconducting behavior compared to samples intercalated by other methods. The stoichiometry of the samples was independently determined by UHV-Raman. This allows us to establish a precise relation between stoichiometry and ρ­(*T*), in particular the *T* at which ρ vanishes. We estimate the transition temperatures from the onset of vanishing resistance (*T* ^onset^) and the vanishing resistance (*T* ^zero^). *T* ^onset^ is the temperature of the local resistivity maximum and *T* ^zero^ is the temperature, at which the *d*ρ­(*T*)/*dT* < 5 × 10^–4^ mΩ·cm K^–1^ in the temperature window shown in Figure 2f. We find that *T* ^onset^ is weakly dependent on *x*, while *T* ^zero^ has a strong *x* dependence. We rationalize this in terms of the Aslamazov–Larkin (AL) model^37^ and Berezinskii–Kosterlitz–Thouless (BKT) fluctuations.^38,39^ The AL model was previously used to describe the excess conductivity of K_3_C_60_ ^19^ and the temperature regime above the critical temperature. We associate this regime with the region around the local maximum in ρ­(*T*), in which ρ­(*T*) depends on the sample dimensionality. The BKT fluctuations between temperatures *T* ^zero^ and the critical temperature are sensitive to defects due to the incomplete stoichiometry for *x* ≠ 3 and may explain the large *x* dependence of *T* ^zero^. In the present experiment, the superconducting transition broadens due to BKT fluctuations, which can be enhanced in thin films by disorder, inhomogeneity in the stoichiometry, Coulomb interactions, and surface scattering in systems with reduced dimensionality. Since the transition width also indicates the defect density, it helps to assess how far the stoichiometry is away from the optimal Rb_3_C_60_. In the present data, we observe the largest broadening for Rb_2.71_C_60_ and the sharpest transition for Rb_2.98_C_60_ in agreement to what is expected from BKT theory. We investigated Rb_3_C_60_ films of 12 and 30 nm thickness (Figure S5) and found that normal-state and the superconducting response show a clear thickness dependence, consistent with bulk transport. Figure 2g depicts ρ­(*x*) at *T* = 300 K for stoichiometries *x* in between *x* = 2.2 and *x* = 3 with Rb_3_C_60_ at the resistance minimum.^40^ There is also a residual conduction of electricity through the Rb network in between the grains. The residual conduction acts as a current path in parallel to the conduction through the doped C_60_ film. The resistance minimum is not affected by the parallel channel because the resistance of the Rb network is almost ten times higher than the resistance of the doped C_60_ film (see Figure S6). The Raman spectra and ρ­(*T*) for each data point in Figure 2g are shown in the Supporting Information (Figure S7).

### Current-Driven Electro-Intercalation: Experiment and Theory

#### Experimental Observation of Rb^+^ Intercalation

In order to explain the mechanism of electro-intercalation of Rb^+^ into C_60_, we performed additional experiments with intermittent DC, continuous AC, and continuous DC currents applied to a C_60_ film with excess Rb evaporated onto its surface. During electro-intercalation, we also monitored the voltage drop across the sample and determined ρ­(*t*). Compared to dedicated measurements of the resistivity shown in Figure 2, where a current of 1 μA was applied, the ρ­(*t*) data shown here was recorded using a current of 10 μA. The larger current which is dissipated onto a partially intercalated sample and the fact that no compensation of thermoelectric voltages could be performed leads to a higher noise level. Yet, the observed time-dependent trends in ρ are unaffected by the magnitude of the current used which is corroborated by evaluating Raman spectra before and after current application.

Figure 3a shows the Raman spectrum collected before starting the electro-intercalation (at time *t* = 0). From this Raman spectrum, we evaluate the initial stoichiometry as *x* = 2.54. The presence of a Rb_3_C_60_ peak confirms that a fraction of the deposited Rb has already intercalated. Figure 3b depicts ρ­(*t*) measured during an electro-intercalation experiment applying an intermittent 10 μA current for 10 s with a period of 3 h in between two consecutive steps of current application. Figure 3c depicts the AC electro-intercalation experiment at a frequency of 1 Hz. We observe an increase in ρ­(*t*) during the intercalation, indicating that no Rb^+^ is intercalated into the C_60_ grains. The Raman spectrum (Figure 3d) taken immediately after AC electro-intercalation attempt confirms a slight reduction of Rb doping of the sample stoichiometry to *x* = 2.50. We rationalize this observation via a slow deintercalation rate which is independent of an applied current. The next experiment investigates intercalation under continuous DC application (Figure 3e). Here, ρ­(*t*) shows a decrease over time, suggesting the Rb^+^ intercalation into C_60_ grains. The Raman analysis shown in Figure 3f confirms Rb intercalation by showing a clear change in stoichiometry from *x* = 2.50 to *x* = 2.69 within 2.6 × 10^5^ s application of a 10 μA DC. This experiment confirms our earlier data of Figure 2f that the application of a 10 μA current indeed intercalates Rb to C_60_ grains and can be used as a means to fine-tune Rb_*x*_C_60_ stoichiometry at room temperature. We now estimate what fraction of the 10 μA current is ionic. The ionic current required to explain the stoichiometry change Δ*x* ≈ 0.2 in Δ*t* ≈ 2.5 × 10^5^ s (Figure 3d,f) for the intercalation of *N*_C_60__∼5 × 10^14^ fullerene molecules is $I_{Rb^{+}}=e\frac{\Delta x}{\Delta t}N_{C_{60}}\approx 60pA$ (see Supporting Information). This is 5 orders of magnitude lower than the current of 10 μA which was used in the experiment. We thus conclude that the current is mainly electronic.

**Figure 3:**
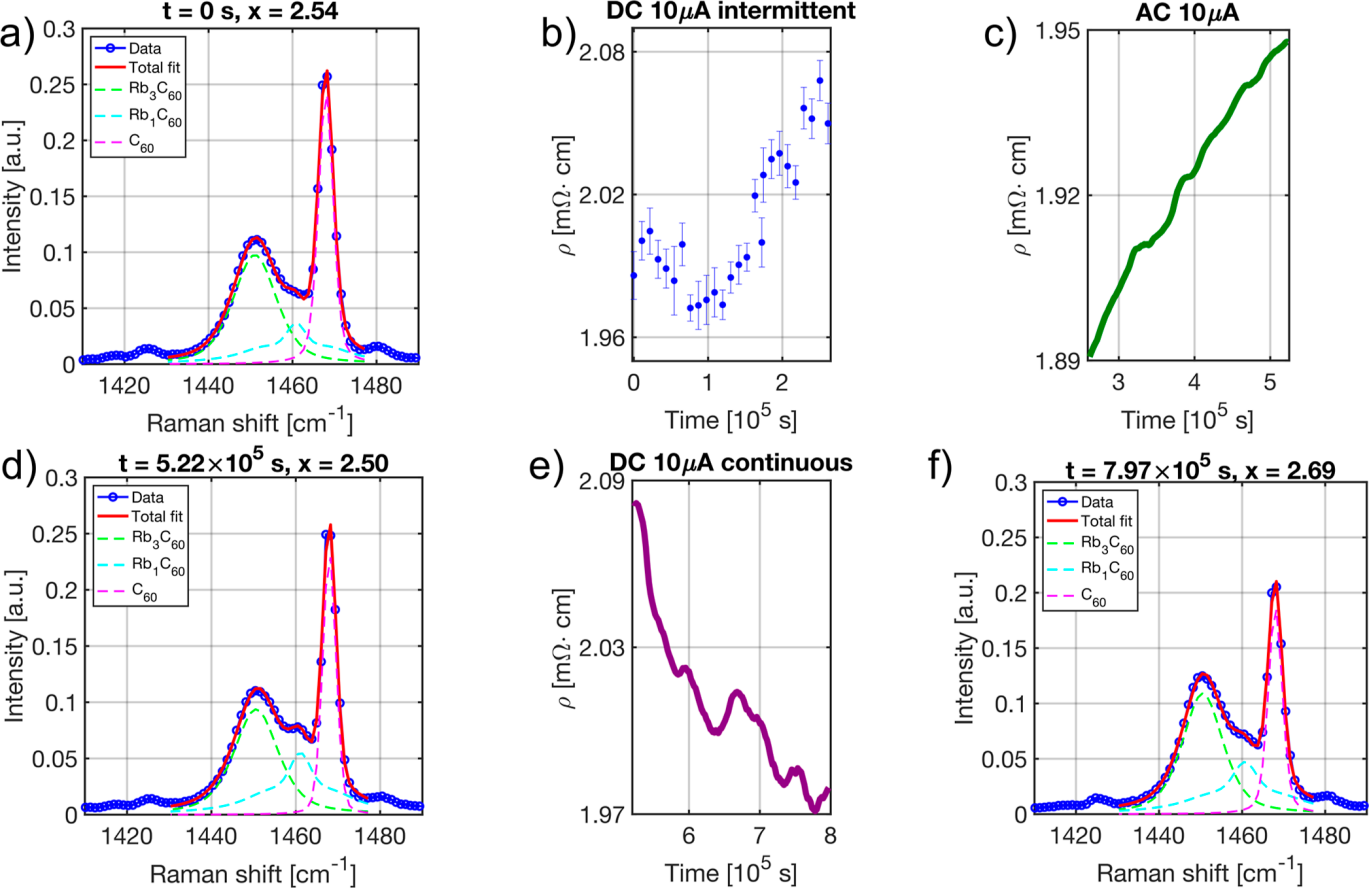
(a) Raman spectrum at *t* = 0 with Rb_2.54_C_60_ stoichiometry. (b,c) Time-dependent resistivity ρ­(*t*) during intermittent DC current (each 3 h for 10 s) and during AC current (1 Hz). (d) Raman spectrum at *t* = 5.22 × 10^5^ s (Rb_2.50_C_60_). (e) ρ­(*t*) during continuous DC current. (f) Raman spectra at *t* = 7.97 × 10^5^ s with stoichiometry Rb_2.69_C_60_. The AC and continuous-DC resistivity traces were smoothed using a moving-median filter with an effective time window of 2 × 10^4^ s and a second-order Savitzky–Golay filter.

#### Theoretical Description of Rb^+^ Electro-Intercalation

We explain the observed electro-intercalation of Rb by three essential processes that describe the intercalation of Rb across the vdW gap to a C_60_ grain (process I), the Rb^+^ diffusion inside a grain of C_60_ and on its surface (process II), and the current-driven Rb^+^ intercalation into C_60_ grains (process III). Process I dominates until the easily accessible Rb has been used up, and process II is very fast but depends on available Rb. The rate-limiting process III is controlled via the applied current and described by the Butler–Volmer model. Processes I–III are summarized and described in Table 1.

**Table 1:** Processes That Describe the Intercalation of Rb^+^ into C_60_ ^a^

process no	process description	process parameter	energy barrier *E*	kinetic role
I	Rb intercalation through the vdW gap between the Rb shell and C_60_	Rb–C_60_ distance	*E* = 0 (vdW distance) *E* ≈ 200 meV (10 Å)	not rate-limiting
II	diffusion of Rb inside the C_60_ grain and on its surface, leading to Rb intercalation of C_60_	diffusion coefficient *D* of Rb inside C_60_	*E* ≈ 200 meV^b^	not rate-limiting
III	tunneling of an electron followed by Rb^+^ intercalation described by Butler–Volmer kinetics	electron tunneling barrier between Rb and Rb_3_C_60_	*E* ≈ 400 meV	rate-limiting and voltage controlled

a Processes I and II describe the intercalation from the vdW Gap and the Rb^+^ diffusion. Process III is the tunneling of an electron followed by the Rb^+^ drift and is controlled via the applied voltage and rate-limiting.

b Estimated for the Rb-poor case.

Process I explains why a fraction of deposited Rb intercalates spontaneously into C_60_ after Rb deposition. If Rb is within the vdW distance to C_60_ (i.e., 4.7 Å), the Rb can intercalate without having to overcome an energy barrier. We have performed calculations of the barriers for Rb intercalation into a C_60_ film with the climbing image-nudged elastic band method (CI-NEB) at three distances: the vdW distance, 10 Å, and 20 Å. For the vdW distance, the intercalation process has no barrier, i.e., it is spontaneous. The barriers for 10 Å and 20 Å are 0.24 and 0.92 eV, respectively (Figure S8). Bader charge analysis along the reaction path shows that the migrating Rb atom is neutral on the left side of the transition state where the driving force arises mainly from the cohesive energy of the Rb slab. It is clear that spontaneous Rb migration will reduce with increasing vdW gap size as all the easily available Rb is used up.

Process II describes diffusion of Rb^+^ on the surface of a C_60_ grain and inside a C_60_ grain. In our calculation, we consider a Rb-poor case where one Rb^+^ is intercalated in an otherwise empty C_60_ lattice and a Rb-rich case where a single Rb vacancy in Rb_3_C_60_ is introduced. The Rb occupies tetrahedral centers of *T_d_* point group symmetry (denoted as *T_d_*) and octahedral sites of *O_h_* point group symmetry (denoted as *O*_*h*_). Our results indicate effective Rb transport via a repeating *O*_*h*_ → *T*_*d*_ → *O*_*h*_ sequence along symmetry-equivalent directions which is more effective than transport via *O*_*h*_ → *O*_*h*_ and *T*_*d*_ → *T*_*d*_ hopping. The calculated energy barrier for the *T*_*d*_ → *O*_*h*_ hop of Rb is ∼0.19 eV for the Rb-poor case and ∼0.49 eV for a vacancy hop for the Rb-rich case (see Figure S9). The energy of the *O*_*h*_ site is about 0.05 eV higher than the *T*_*d*_ site. For the energy barrier in the Rb-rich case, we obtain a diffusion constant *D* ≈ 4 × 10^–15^ m^2^/s at 300 K. The diffusion time is estimated as *t* = *L* ^2^/(2*dD*) with dimensionality *d* = 1 for thickness-limited diffusion and a film thickness *L* = 15 nm as *t* ≈ 10^–2^ s. Hence, *t* is negligible compared to the observed time scales for the intercalation. Processes I and II provide fast local equilibration of available Rb^+^ within and around individual grains. However, this easily available Rb is generally not sufficient to synthesize Rb_3_C_60_. As we will show in the following, an applied bias is required to reach Rb_3_C_60_ stoichiometry.

In process III, the intercalation of Rb^+^ into a C_60_ grain proceeds via the Butler–Volmer mechanism in the presence of an applied current. This process is rate-limiting and sets the multiday time scale of the experiment. If a current is applied to a network of connected C_60_ grains surrounded by Rb, there is a voltage drop across each C_60_ grain. This is similar to metal nanowire networks where a large voltage drop inside the film of interconnected nanowires has been observed.^36^ Due to the voltage drop, the barrier for electron tunneling from the Rb metal shell into the C_60_ grain is reduced. An electron that has tunneled into the C_60_ grain can attract a Rb^+^ ion. In the following, we estimate the tunneling barrier. The cross section of the heterostructure is Rb/Rb_3_C_60_/C_60_/Rb_3_C_60_/Rb. Process III involves three subprocesses 1–3 that are depicted in detail in Figure 4a. The first subprocess is an electron tunneling from the Rb metal on the left contact into the center of the C_60_ grain. The second subprocess is a Rb^+^ ion being attracted by Coulomb force from the surrounding Rb to the C_60_ grain. Due to the presence of an electron inside the C_60_ core, the Rb^+^ ion that detaches from the Rb metal on the right side has to overcome only a small van der Waals barrier.^41^ The third subprocess is that an electron flows from the right contact to the voltage source and closes the circuit. Let us consider the tunneling subprocess 1 in more detail. The tunneling gap between the Rb metal and the Rb_3_C_60_ grain is indicated in Figure 4a. In Figure 4b, we consider the band diagram across a partially intercalated C_60_ grain of total stoichiometry *x* = 2.50. The band diagram was constructed based on the literature values of electron affinity of C_60_, χ_C_60__ = 2.7 eV,^42^ the work function of Rb, ϕ_Rb_ = 2.1 eV,^43^ the work function of Rb_3_C_60_ is taken as $\phi_{Rb_{3}C_{60}}=2.7$ eV, the HOMO–LUMO gap of C_60_ is taken to be E_g_ = 2.3 eV,^44^ and the Fermi level of C_60_ is located 0.9 eV below the LUMO, giving a work function ϕ_C_60__ = χ_C_60__ + 0.9 = 3.6 eV.^45^ In order to evaluate the band bending of C_60_ at the interface to Rb_3_C_60_, we consider the Fermi levels of isolated C_60_ and Rb_3_C_60_ with a common vacuum level. The Fermi level of Rb_3_C_60_ lies at higher energy than the Fermi level of C_60_ and electrons flow from Rb_3_C_60_ to C_60_. In the present system of a partially intercalated grain, there is a common Fermi level across the interface between the Rb_3_C_60_ shell and the C_60_ core. Hence, when electrons are transferred from Rb_3_C_60_ to C_60_, the bands of C_60_ bend downward close to the interface. In Figure 4b, the band bending can be seen. The voltage drop is chosen such that the left contact is at negative potential w.r.t. the right contact.

**Figure 4:**
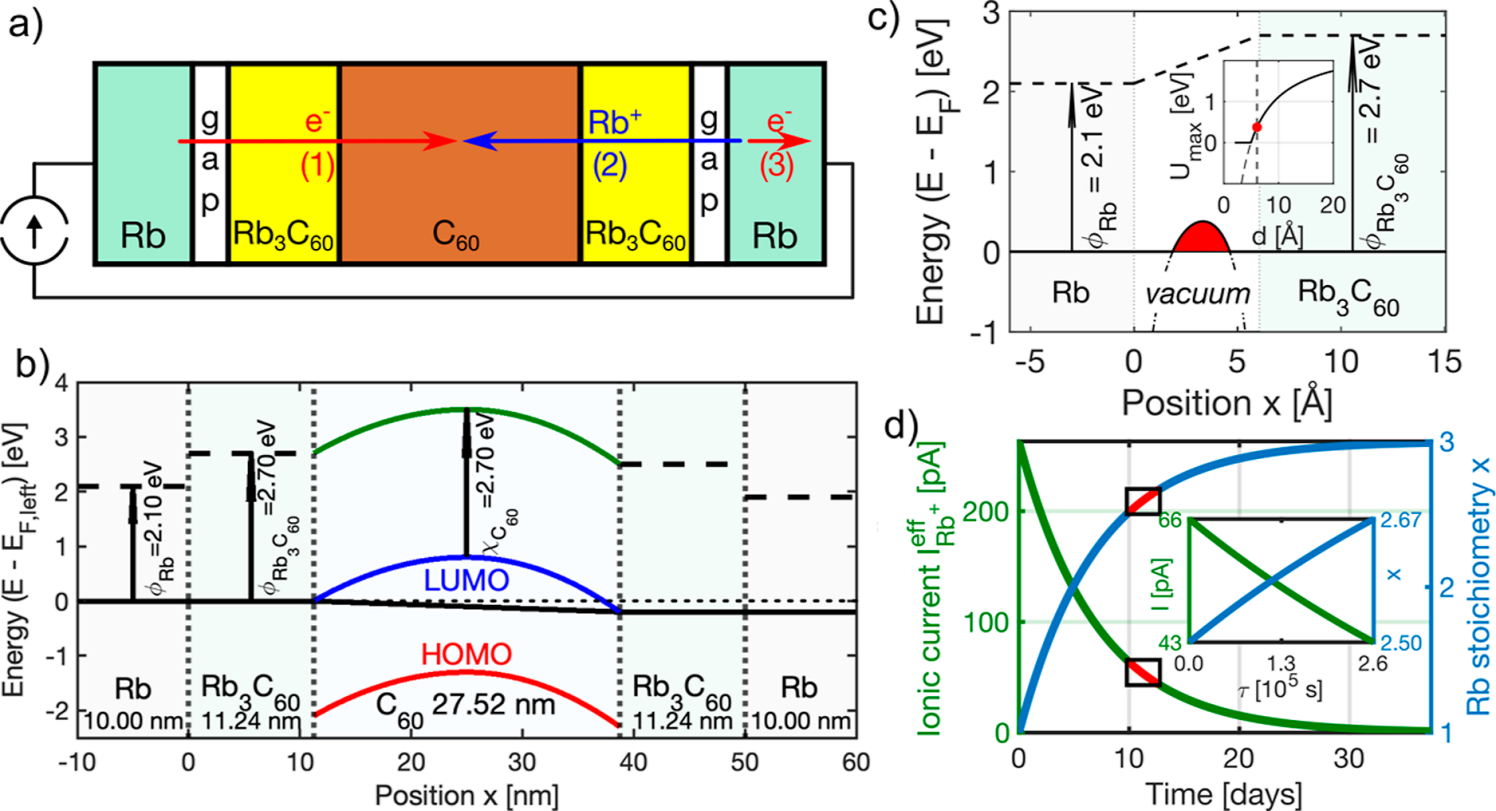
(a) Electro-intercalation of Rb^+^ from outside the vdW distance. Sketch of cross section across a core–shell structure (Rb outside, Rb_3_C_60_ inside). Electron tunneling and Rb^+^ motion are indicated. (b) Band diagram of a Rb/Rb_3_C_60_/C_60_/Rb_3_C_60_/Rb heterostructure. Electron tunneling through the gaps occurs along the direction of increasing *x*. (c) Calculated effective tunneling barrier between Rb and Rb_3_C_60_. The inset denotes the barrier height versus the Rb–Rb_3_C_60_ separation. Details of the calculations are provided in Figure S10 of the Supporting Information. (d) Calculated ionic current according to the Butler–Volmer model along with the stoichiometry change (right vertical axis). Rectangles indicate the time span probed experimentally. The inset shows the experimentally relevant stoichiometry change versus time.

Figure 4c depicts the effective tunneling barrier for the electron tunneling between two quantum wells resembling the Rb metal and Rb_3_C_60_ with a vacuum gap in between (see Figure S10 of the Supporting Information). For the calculation of the tunneling barrier, we considered the Volta potential and effect of image charges. The Rb metal and Rb_3_C_60_ are modeled as quantum wells, and bringing them closer together results in a reduction of the tunneling barrier that the electron needs to overcome when tunneling from the Rb metal into Rb_3_C_60_. For a distance of 6.06 Å, we calculated an effective barrier height equal to ∼0.4 eV. The inset of Figure 4c shows the barrier as a function of Rb–Rb_3_C_60_ distance. Let us now calculate the ionic Rb^+^ current using the Butler–Volmer model. This model provides the interfacial ionic current density *j*(η) = *e*Γ_s_[*k*_f_(η) – *k*_b_(η)], where *k*_f_, *k*_b_ are forward/backward rate constants, Γ_s_ is the effective number of sites on the C_60_ surface (here the surface density of C_60_ Γ_s_ ≈ 2/nm^2^), η is the overpotential, and *e* is the elementary charge. Denoting l = 50 nm the typical size of the grains of C_60_ and Δ*V* the total voltage drop, we estimate η = ( l /*L*)­Δ*V* ≪ *k*_B_ *T*/*e*. Using transition-state theory (TST) with transmission factor κ ≈ 1 for a small overpotential η, we get the interfacial ionic current by unit of the Rb/Rb_3_C_60_ interface, which we model as a section of the device for each layer of grains, that is, *wL* × *L*/ l . The total current in the Butler–Volmer model reads

$$I_{Rb^{+}}\approx \frac{\kappa wL\Gamma_{s}e^{2}\Delta V}{2\pi \hbar }\exp\left(-\frac{\Delta G_{0}^{‡}}{k_{B}T}\right)\exp\left(-2\kappa_{tun}d\right)$$ 1

Here, Δ*G*_0_^‡^ is the energy barrier which corresponds to the energy required for promoting the electron into the top of the C_60_ LUMO. The last term is a tunneling rate of the electron throughout the gap of length *d* with transmission of $\kappa =\sqrt{2mU}/\hbar$ , where *U* is the effective barrier and *m* is the free electron mass. In order to make eq 1 time-dependent, we model the effective number of C_60_ sites with a phenomenological Avrami model. This model is generally applicable for all the A_*x*_C_60_ material system.^35^ Using a current $I_{Rb^{+}}$ that does not depend on the grain size, we can extract an effective ionic current:

2 $$I_{Rb^{+}}^{eff}\left(t\right)=e\frac{dx\left(t\right)}{dt}N_{C_{60}}=I_{Rb^{+}}\cdot \exp\left(-\frac{I_{Rb^{+}}}{3eN_{C_{60}}}t\right)$$

Figure 4d depicts the plot of $I_{Rb^{+}}$ as a function of the intercalation time *t*. Here, we used Δ*G*_0_^‡^ = 0.55 eV, *U* = 0.5 eV, and *d* = 0.65 nm. Overall, the predicted intercalation dynamics agrees well with the experimental results. In the Supporting Information, we discuss the roles of electromigration and field-induced Rb ionization in Rb^+^ intercalation.

### Electron–Phonon Coupling in Rb_3_C_60_ Thin Films

When intercalating Rb^+^ into the C_60_ crystal, the electronic structure changes to a metal with high density of states at the Fermi level which boosts electron–phonon coupling (EPC). The EPC of Raman active Rb_3_C_60_ phonons can be determined by Raman spectroscopy from the full width at half-maximum (FWHM) increase when going from C_60_ to Rb_3_C_60_. In the presence of EPC, the phonon lifetime is reduced, which leads to an increase of the FWHM in Rb_3_C_60_.^25,46,47^ Therefore, measuring the increase in the Raman mode’s FWHM upon changing C_60_ to Rb_3_C_60_ allows one to determine the EPC constant λ_*i*_ of a particular phonon.^25,46^ There are 37 Raman active phonons in C_60_,^48^ and FWHM Raman analysis has been used to determine the dimensionless EPC constant λ_R_ ^49^ given by

3 $$\lambda_{R}=\sum_{i}\lambda_{i}=\sum_{i}\frac{d_{i}}{\pi N\left(E_{F}\right)}\frac{\Delta \Gamma_{i}}{\omega_{i}^{2}}$$

In eq 3, ω_*i*_, *d*_*i*_, and ΔΓ_*i*_ are the unrenormalized discrete phonon frequency, mode degeneracy, and mode broadening for the *i*-th mode, respectively. *N*(*E*_F_) is the DOS at the Fermi level, which is equal to *N*(*E*_F_) = 15 states/eV for Rb_3_C_60_.^25^ Previous works found a dominant contribution of the H_g_(2) phonon mode which contributes λ­[H_g_(2)] ≈ 0.2,^25,46^ and a total EPC constant for all Raman active modes λ_R_ ≈ 0.5 was claimed for thick Rb_3_C_60_ films.^25^ The contributions to the FWHM of Raman modes are EPC, phonon–phonon scattering, and defect scattering. The effects of phonon–phonon scattering depend on temperature, and we find only minimal change in the FWHM of H_g_(1) and H_g_(2) phonons at 5 and 300 K. The defect scattering contribution is minimized by using phase-pure Rb_3_C_60_ in our experiment. In addition to Raman spectroscopy, EPC can also be determined from *d*ρ/*dT*, the slope of the resistivity with temperature. This determination of EPC includes all phonons (also Raman inactive phonons) whose energy is less than *k*_B_·*T* (*T* is the temperature in which the linear slope is observed and *k*_B_ the Boltzmann constant).^50,51^ Thus, the higher energy optical phonons observed in Raman do not contribute to the EPC constant measured in transport. To highlight the difference to λ_R_, we introduce λ_tr_, the EPC determined from *d*ρ/*dT*. Previously, λ_tr_ was determined in the temperature range between 100 K and 350 K at constant volume from the measured slope *d*ρ/*dT* ^50,51^ according to

4 $$\lambda_{tr}=0.246{\left(\hbar \omega_{p}\right)}^{2}d\rho /dT$$

In eq 4, ℏω_p_ (the plasma energy) is to be taken in units of eV and *d*ρ/*dT* is to be taken in units μΩ·cm/K. The reported values for ℏω_p_ from literature have a wide range, e.g., ℏω_p_ = 0.6 eV (optical spectroscopy)^52^ and ℏω_p_ = 1.2 eV (theory).^53^ Using *d*ρ/*dT* ∼2 μΩ·cm/K and ℏω_p_ = 1.2 eV, λ_tr_ = 0.65 was obtained.^50^ The EPC constant enters the McMillan formula for the critical temperature *T*_C_,^54^ which reads

5 $$T_{c}=\frac{\hbar \omega_{\ln}}{1.2k_{B}}\exp\left(-\frac{1.04\left(1+\lambda \right)}{\lambda -\mu^{*}\left(1+0.62\lambda \right)}\right)$$

For Rb_3_C_60_, μ* = 0.1 ^22^ and an average phonon frequency ω_ln_ = 1300 cm^–1^ from theory^49^ have been used.^25^ For λ_R_ = 0.5, a value of *T*_C_ = 17 K was predicted^25^ (the actual *T*_C_ of the Rb_3_C_60_ sample from transport was *T*_C_ = 28 K).^25^ There is a discussion^26,55–58^ if the McMillan formula is applicable in the case of Rb_3_C_60_ because the bandwidth of the electron energy bands is close to the phonon energy and correlation effects may play a role. Usually, the inclusion of correlation effects lowers *T*_C_, but in the case of fullerides, correlation effects and EPC can cooperate to increase *T*_C_.^59,60^ No effective theory exists that predicts *T*_C_ of Rb_3_C_60_ in the presence of correlations and EPC.

Let us now determine EPC in Rb_3_C_60_ from Raman and transport measurements. We have synthesized a superconducting Rb_3_C_60_ film in situ and measured ρ­(*T*), from which we extracted the transition temperatures *T* ^zero^ and *T* ^onset^ and the slope *d*ρ/*dT*. We compared the observed transition temperatures to the critical temperatures estimated from EPC constants obtained from Raman and transport. Figure 5a shows Raman spectra of C_60_ and Rb_3_C_60_ in the wide energy range. We employed eq 3 to determine EPC from the FWHM of each Raman active phonon mode (Figure S11). Overall, our FWHM are in good agreement to previously published works.^46,47,61^ In Figure 5b, we plot the contribution of each Raman active phonon mode to the EPC constant λ_R_. It can be seen that, in particular, the H_g_(2) phonon mode has a large contribution to the total λ_R_, in agreement to previous works using Raman spectroscopy on Rb_3_C_60_ ^25,46,47^ and a recent ARPES study on K_3_C_60_.^62^ We obtain a total EPC from Raman λ_R_ = 0.334 from which we estimate *T*_C_ via eq 5 to obtain *T*_C_ = 0.78 K which is considerably lower than the experimental value of *T*_C_ (c.f. Figures 2f and 5c). For estimating *T*_C_, we have used an experimental value ω_ln_ = 441 cm^–1^ which is lower than the previously used ω_ln_.^25^ We explain our lower value of ω_ln_ as follows. In the calculation of ω_ln_, each phonon mode is weighed with its EPC constant,^49^ and because of the dominance of *H*_g_(2) EPC, the resulting ω_ln_ is small in comparison to an ω_ln_ weighted by similar EPC constants. The transport EPC λ_tr_ is determined from the experimental slope whose fit is shown in Figure 5c yielding *d*ρ/*dT* = 0.827 μΩ·cm/K which is a factor ∼2 lower than in previous experiments.^50^ The previous experiment^50^ was carried out at constant sample volume. The volume of the present thin film sample is constrained by the Si substrate with a ∼10 times lower linear thermal expansion coefficient than Rb_3_C_60_ ^63^ making the present and previous experiments comparable. From *d*ρ/*dT*, we obtain a value of λ_tr_ = 0.293, and from eq 4, we obtain *T*_C_ = 0.71 K. We consider the similarity of λ_R_ and λ_tr_ coincidental because different phonons contribute to λ_R_ and λ_tr_. We investigated the superconducting transition of the same sample used for Raman and dρ/*dT* measurements. Figure 5c depicts ρ­(*T*) and *d*ρ/*dT* yielding *T* ^onset^ = 24.5 K and *T* ^zero^ = 9.2 K which is in disagreement to the estimation of *T*_C_ from λ_R_ and λ_tr_. The following three points are important when discussing this disagreement. First, not all Rb_3_C_60_ phonons are Raman active and λ_R_ and hence *T*_C_ are underestimated. Second, in λ_tr_, only low-energy phonons contribute whose energy is below the linear *d*ρ/*dT* range. The above two points explain the underestimation of *T*_C_ by considering either λ_R_ or λ_tr_. It is thus likely that both λ_R_ and λ_tr_ contribute to the total EPC. Third, if correlation effects are present and enhance EPC,^59,60^ we underestimate *T*_C_ from our EPC analysis.

**Figure 5:**
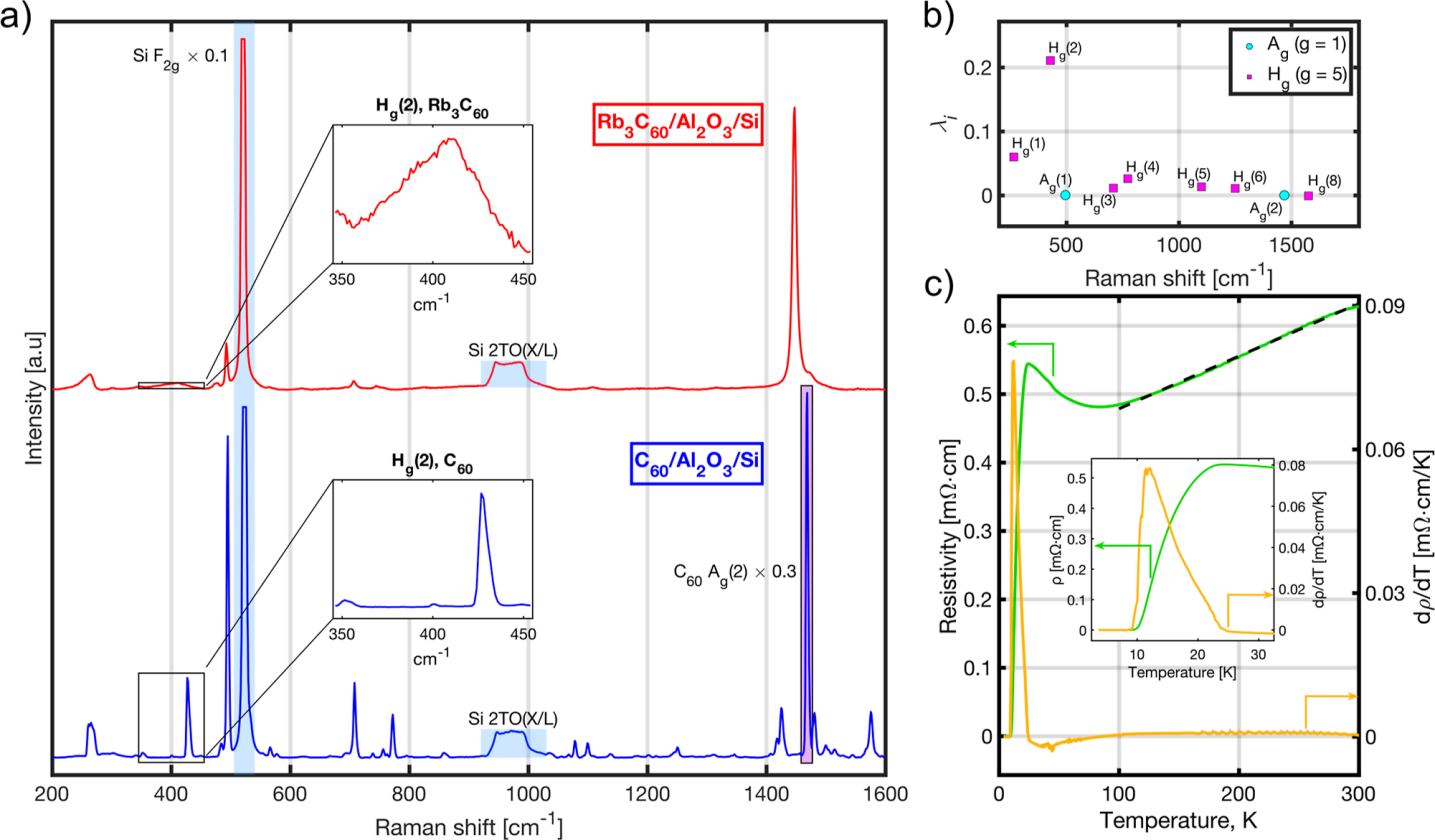
(a) Raman spectra of C_60_/Al_2_O_3_/Si and Rb_3_C_60_/Al_2_O_3_/Si. The insets show the H_g_(2) peaks with a large electron–phonon coupling (EPC) constant in the case of Rb_3_C_60_. The total EPC of this sample was λ_R_ = 0.334. (b) EPC constant of individual phonon modes versus the phonon energy. (c) Rb_3_C_60_ temperature dependence of resistivity and its derivative *d*ρ/*dT*. In the linear regime, we obtain *d*ρ/*dT* = 0.827 μΩ·cm/K yielding an EPC constant λ_tr_ = 0.293 and *T*_C_ = 0.71 K. The superconducting transition of this sample is characterized by *T* ^zero^ = 9.2 K and *T* ^onset^ = 24.5 K.

## Conclusions and Outlook

We have introduced electro-intercalation as a method for on-chip intercalation chemistry where ions are driven by electronic currents. Electro-intercalation can be used to control the stoichiometry in order to fine-tune phase transitions. In the present work, we have controlled the superconducting phase transition in fullerides by Rb^+^ intercalation. Alkali metals with a low work function are particularly suited for electro-intercalation because the corresponding barriers for electron tunneling are small. We conclude that electro-intercalation works well for a nanostructured C_60_ film that consists of grains because such a film provides a high density of Rb/Rb_3_C_60_/C_60_ interfaces. An applied current leads to a potential drop over a C_60_ grain which facilitates electron tunneling into the C_60_ grain followed by the intercalation of a Rb^+^ ion. The exciting aspects of this experiment are its high level of stoichiometric precision, ultimate cleanliness ensured by UHV conditions, and spatially homogeneous doping. Moreover, there is no lower limit for the volume of the host material that can be intercalated. This doping method could also be applied to the on-chip synthesis of fulleride superconductor heterostructures. In particular, topological insulator (TI)-fulleride interfaces^9^ are interesting candidates for superconducting fulleride growth on the TI and can probably be synthesized by electro-intercalation. Electro-intercalation also allows, in principle, for the synthesis of ternary fulleride superconductors such as Rb_2_CsC_60_ with a higher critical temperature^24^ or polymeric Mg_4_C_60_.^28^ The presented experimental setup can also be used for experiments on light-induced superconductivity in Rb_3_C_60_, where the four-point resistance measurement is carried out during light irradiation. Future experiments could also include a microfabricated on-chip Rb or Cs ion source such as the on-chip Yb source built recently.^64^ This ion source would allow for shuffling ions into the sample region. A future theoretical work could consider the unified description of processes I–III by a single theoretical method. We expect that electro-intercalation works not only in C_60_ but also in a large variety of organic materials such as picene, phenanthrene, coronene, graphene nanoribbons, and metal–organic frameworks and could be used to search for and identify new alkali metal-doped superconducting materials.

## Experimental Section

### Sample Preparation

In the first step (Figure 1b II), a C_60_ film was deposited on the Al_2_O_3_ (sapphire) substrate by evaporation from a quartz crucible resistively heated to 400 °C. The base pressure in the UHV system was *p* ∼ 2 × 10^–10^ mbar. The deposition rate was 5 Å/min monitored and controlled using a quartz crystal microbalance (QMB). During film growth, the substrate was kept at 120 °C, followed by postannealing at 200 °C for 5 min. Hereafter, the C_60_ sample was immediately checked by Raman spectroscopy in the range of the strong A_g_(2) Raman mode of C_60_, observed at 1456 cm^–1^ for C_60_ at temperatures *T* > 260 K and 1467 cm^–1^ at *T* < 260 K.

In the next step, Rb doping of C_60_ was performed by evaporation of Rb from a well-degassed getter source. The Rb flux, calibrated using a QMB, was approximately 0.1 Å/min and simultaneously monitored throughout the doping process. As the evaporation was carried out with the sample mounted on a cryomanipulator, the resistance evolution could be recorded in situ, enabling the identification of the Rb_3_C_60_ phase by the characteristic resistance minimum.^40^ During doping, Raman spectra were simultaneously acquired, focusing on the evolution of the A_g_(2) mode, which progressively shifted from the Rb_1_C_60_ position at 1458 cm^–1^ toward the single Rb_3_C_60_ peak at 1449 cm^–1^. The doping was continued until the spectrum exhibited only the Rb_3_C_60_ feature, indicating completion of the intercalation process. In order to resolve these peaks, all these Raman acquisitions were performed at temperatures less than 10 K.

### Raman Spectroscopy and Mapping

Raman spectroscopy was performed using λ_e*x*t_ = 532 nm excitation wavelength with a laser power *P* ≈ 10 mW. The cryomanipulator in our experimental setup was equipped with two encoded stepper motors. It allowed automated sample translations in the plane perpendicular to the laser beam. The minimal stepsize for translation was 250 nm. The mapping spatial resolution was limited by the laser beam spotsize arising from excitation wavelength λ_e*x*t_ and numerical aperture NA of the objective, which in our case was estimated from the Airy pattern diameter as follows: *d* ≈ 1.22λ_e*x*t_/*NA* ≈ 1.18 μm.

### Electrical Transport Characterization

Electrical transport measurements were performed in a van der Pauw geometry with electrical contacts precisely placed at the four corners of squared substrate. The temperature-dependent resistivity and superconducting transition were measured with a precision current source Keithley 6220 and nanovoltmeter Keithley 2182A operated in Delta mode to suppress spurious thermoelectric voltages. The Delta acquisition frequency was set at 1 Hz, and the magnitude of the excitation current was 1 μA, ensuring negligible self-heating of the sample in the superconducting state.

During the electro-intercalation process, the sample resistance was continuously monitored using a Sourcemeter Keithley 2400 operating in a DC mode with a constant current of 10 μA, while the voltage drop across the film was recorded simultaneously. Electrical transport measurements also allow for the verification of the film homogeneity by interchanging the current and voltage contact pairs and by rotating the measurement configuration by 90°, as permitted by the van der Pauw geometry. Considering the strict square substrate geometry and the accurate placement of the contacts in the corners, the variation in the measured resistances of all configurations did not exceed 5%.

### Rb_*x*_C_60_ Stoichiometry Estimation Using Raman Spectra

We determine the Rb_*x*_C_60_ stoichiometry from the A_g_(2) Raman peak components corresponding to C_60_, Rb_1_C_60_, and Rb_3_C_60_. For the analysis, we assume that the number of C_60_ molecules inside the laser spot is conserved during Rb intercalation. The peak areas of components C_60_, Rb_1_C_60_, and Rb_3_C_60_ in the *i*-th doping step are denoted by $A_{i}^{C_{60}}$ , $A_{i}^{Rb_{1}C_{60}}$ , and $A_{i}^{Rb_{3}C_{60}}$ , respectively. Then *x*_*i*_, the stoichiometry in the *i*-th doping step, is given by

6 $$x_{i}=\alpha \cdot \frac{A_{i}^{Rb_{1}C_{60}}}{A_{0}^{C_{60}}}+3\beta \cdot \frac{A_{i}^{Rb_{3}C_{60}}}{A_{0}^{C_{60}}}$$

The parameters α and β are determined from a regression analysis as α = 5.84 and β = 2.95, respectively (Supporting Information). The relative Raman cross sections for C_60_, Rb_1_C_60_, and Rb_3_C_60_ are equal to 1, 1/α, and 1/β, respectively. Let us consider a general sample which has three Raman peaks and consists of C_60_, Rb_1_C_60_, and Rb_3_C_60_ fractions with relative Raman cross sections of 1, 0.17, and 0.34, respectively. We assume that the error bar of the Raman peak corresponding to each phase is given by the noise of the Raman spectrum. From the experimentally observed noise, we estimate at most 10% error for the Raman intensities (see Figure 2f). Considering a stoichiometry *x* close to *x* = 3, we typically have an intense Raman peak corresponding to Rb_3_C_60_ and a weaker or no Raman peak corresponding to C_60_. The cross section of C_60_ is ∼3 times higher than the cross section of Rb_3_C_60_. Therefore, if we have a Rb_3_C_60_ sample and see no C_60_ peak, it means the error bar of the stoichiometry *x* = 3 is about 10%/3 ≈ 3%. For mixed phases, the error can be slightly higher because of the contributions of all three peaks to the total error.

### Quantum-Chemical Calculations

Density functional theory (DFT) calculations were performed using the Vienna Ab initio Simulation Package (VASP) version 6.4.2.^65,66^ Interactions between ion cores and valence electrons were treated using the projector-augmented wave (PAW) method.^67,68^ Exchange–correlation effects were described using the Perdew–Burke–Ernzerhof (PBE) generalized gradient approximation (GGA) functional.^69^ The Rb_sv_ PAW potential with a valence electron configuration of 4s^2^4p^6^5s^1^ and the standard C PAW potential with a valence electron configuration of 2s^2^2p^2^ were used. A plane-wave energy cutoff of 500 eV and a 2×2×2 Monkhorst–Pack *k*-point mesh were applied. Structural relaxations were converged to 10^–5^ eV in total energy and 0.01 eV·Å^–1^ in forces. Reference structures for pristine C_60_ and fully intercalated Rb_3_C_60_ were obtained by relaxing the Fm3̅m cubic phase. The optimized lattice constant was 14.797 Å for C_60_ and 15.104 Å for Rb_3_C_60_ (∼2% expansion upon intercalation). Using the relaxed Rb_3_C_60_ structure, minimum-energy migration pathways for isolated Rb^+^ hopping and vacancy migration between the octahedral (*O*_*h*_) and tetrahedral (*T*_*d*_) pockets were calculated with the climbing-image nudged elastic band (CI-NEB) method,^70^ thereby providing the bulk migration barriers. To investigate Rb transport across the Rb–C_60_ interface, slab supercells were constructed containing a Rb slab in contact with two C_60_ layers. Three interface geometries were considered, including vdW-vdW, vdW-10 Å, and vdW-20 Å, to compare the Rb migration at vdW-sized and large imposed interfacial gaps. Two other interface geometries, 10 Å-10 Å and 20 Å-20 Å, were also constructed to determine the energy barrier for Rb migration across the 10 Å and 20 Å interfacial gaps. The in-plane lattice constants were fixed to those of the relaxed Rb^+^ structure, and a sufficiently large out-of-plane cell dimension was used to preserve the intended gap sizes. During relaxation, the middle layer of the Rb slab was held fixed to mimic bulk-like behavior of the Rb nanowire. All numerical settings for the slab calculations were identical to those used for the bulk systems, except that a Γ-centered 4 × 4 × 1 *k*-point mesh was employed and a 0.02 eV·Å^–1^ force tolerance was used during relaxations and CI-NEB calculations. CI-NEB was then applied to obtain minimum-energy paths and transition states (TS) for Rb migration across various interfacial gaps. Bader charge analysis^71^ was used to quantify the charge state of the migrating Rb atom along each CI-NEB pathway.

### Estimate of the Rb^+^ Diffusion Coefficient and Intercalation Time

To obtain a realistic estimate of the Rb^+^ diffusion prefactor and diffusion coefficient in the Rb-rich lattice, we complemented the DFT CI-NEB barriers with finite-temperature molecular dynamics using Grimme’s extended tight-binding (xTB) Hamiltonian,^72,73^ as implemented in the DFTB+ code.^74,75^ The Rb_3_C_60_ Fm3̅m structure (four C_60_ and 12 Rb per cell) was first reoptimized at the xTB level, and NVT dynamics at *T* = 300 K were carried out with a Nose–Hoover thermostat for 5 ps simulation time and using a 1 fs time step. Selected Rb ions that remained localized in either *O*_*h*_ or *T*_*d*_ sites were monitored and their velocities projected onto the geometric hop direction between neighboring *O*_*h*_ and *T*_*d*_ pockets. From the velocity autocorrelation function and its Fourier transform, we extracted characteristic vibrational frequencies along the hop direction and, by multiplying with the coordination numbers of the corresponding sites, effective attempt frequencies.

## Supplementary Material



## Notes

Photograph of the sample inside UHV through the inverted flange; regression analysis for stoichiometry determination by Raman spectroscopy; individual Raman spectra of Raman maps; line shape analysis for stoichiometry determination; thickness-dependent conductivity for a 12 and 30 nm Rb_3_C_60_ film; resistance of the residual Rb channels; stoichiometry determination and ρ­(*T*) for each stoichiometry *x*; calculation of the vdW intercalation barrier; Bader charge analysis; calculation of the diffusion barrier for Rb inside C_60_; calculation of intercalation time; details for the Butler–Volmer model; calculation of the tunneling barrier between Rb and Rb_3_C_60_; and determination of FWHM of Raman modes in C_60_ and Rb_3_C_60_ (PDF)
