# Supplementary material for: On-Chip Tuning of Superconductivity in Fullerides via Current-Driven Rb+ Intercalation
Source: ACS Nano. 2026 Jun 9;20(24):17360–72. doi: 10.1021/acsnano.6c02466 (PMC13296603; doi:10.1021/acsnano.6c02466)
Supplement: Supplementary file 1 [file nn6c02466_si_001.pdf]

Supporting information for: On-chip tuning of superconductivity in fullerides via current-driven Rb^+ intercalation

Konstantin P. Shchukin,*,†,‡ Oliver N. Gallego Lacey,¶ Baptiste Coquinot,§ Jacek Jakowski,‖ Jingsong Huang,⊥ Patrik Staudenmayer,† Yannic Falke,‡ Ram Prakash Pandeya,† and Alexander Grüneis*,†

†*Institut für Festkörperelektronik, Technische Universität Wien, Gußhausstraße 25, Vienna, 1040, Austria*

‡*II. Physikalisches Institut, Universität zu Köln, Zülpicher Strasse 77, Cologne, 50937, Germany*

¶*CEA, Universite Grenoble Alpes, IRIG-Pheliqs, Grenoble, 38000, France*

§*Institute of Science and Technology Austria (ISTA), Am Campus 1, Klosterneuburg, 3400, Austria*

‖*Computational Sciences & Engineering Division, Oak Ridge National Laboratory, Oak Ridge, Tennessee, 37831, USA*

⊥*Center for Nanophase Materials Sciences, Oak Ridge National Laboratory, Oak Ridge, Tennessee, 37831, USA*

E-mail: konstantin.shchukin@tuwien.ac.at; alexander.grueneis@tuwien.ac.at

Raman mapping and Raman lineshape analysis

The Raman maps in Figure 2 of the manuscript were performed by scanning the laser spot on the sample within a defined region. This was achieved via moving the cryomanipulator by encoded motors. The zero position was aligned with respect to the sample edge. Figure S1 depicts a photograph of the sample edge that was taken through the objective of the inverted flange when the sample was in UHV. A 5x objective was used for taking this photograph.

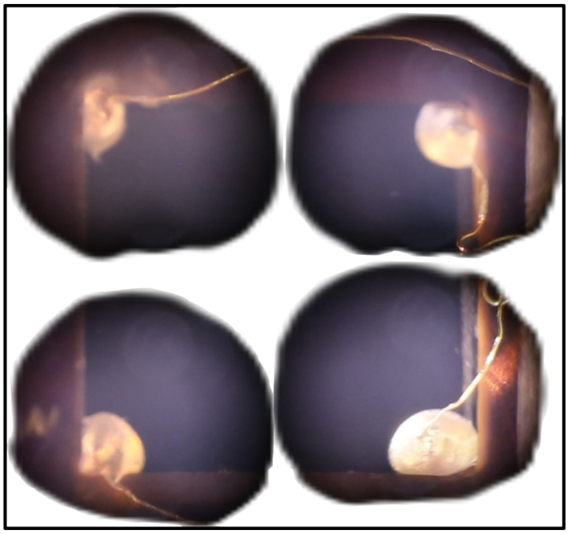

Figure S1: Photograph through the microscope objective through the inverted flange shown in Figure 1 of the main paper.

Estimation of Rb_xC_{60} stoichiometry by Raman spectroscopy

To estimate the stoichiometry of Rb_xC_{60} compounds synthesized with increasing Rb doping level, we developed an approach based on the measured Raman intensities of C_{60}, Rb_1C_{60} and Rb_3C_{60}. In our estimation, we assume that the number of C_{60} molecules within the laser spot stays constant and a transformation from undoped C_{60} to Rb_1C_{60} and Rb_3C_{60} is induced by Rb doping. This estimation is reasonable since the lattice constants of C_{60}, Rb_1C_{60} and Rb_3C_{60} differ by only 2%. That is, the Raman response originates from a weighted combination of three species: pristine C_{60}, Rb_1C_{60}, and Rb_3C_{60}. Upon doping, the

original $A_g(2)$ peak of C_{60} is partially converted to the new peaks of Rb_1C_{60}, and Rb_3C_{60} at lower frequencies. To account for differences in the Raman cross sections of these species, we introduce factors, which we determine by a least squares fit. Once we know the value of the Raman cross sections, we can estimate the stoichiometry x for any Rb_xC_{60} sample from the area of the three Raman peaks after Rb intercalation corresponding to C_{60}, Rb_1C_{60}, Rb_3C_{60} and the area of the Raman peak of the initial C_{60} film prior to intercalation. For example, if we have a pure C_{60} sample and convert that sample into a pure Rb_3C_{60} sample, we can compare the peak areas of the $A_g(2)$ phonon mode of the two samples. A similar situation is true for Rb_1C_{60}. Moreover, for a general sample with mixed contributions from C_{60}, Rb_1C_{60} and Rb_3C_{60} that was made from a pure C_{60} sample, we assume conservation of the number of scatterers (i.e. number of C_{60} molecules). The Raman peak areas of the initial C_{60} peak ($A_0^{C_{60}}$) and the three peaks after doping: $A_i^{C_{60}}$, $A_i^{Rb_1C_{60}}$ and $A_i^{Rb_3C_{60}}$ are collected from Raman measurements carried out before and after doping. Since the number of C_{60} molecules in the laser spot is unchanged by the doping process we write the relation between the initial Raman peak area $A_0^{C_{60}}$ and the three peak areas after doping as

$$A_0^{C_{60}} = A_i^{C_{60}} + \alpha \cdot A_i^{Rb_1C_{60}} + \beta \cdot A_i^{Rb_3C_{60}}. \tag{1}$$

Here we have introduced the correction factors α and β that account for the fact that Rb_1C_{60} and Rb_3C_{60} have a different Raman cross section compared to C_{60}. Note that, $1/\alpha$ and $1/\beta$ are equal to the Raman cross sections of Rb_1C_{60} and Rb_3C_{60}, respectively.

Rearranging the above equation in such a way as to express the change of the Raman intensity of the peak corresponding to undoped C_{60} yields

$$\alpha \cdot A_i^{Rb_1C_{60}} + \beta \cdot A_i^{Rb_3C_{60}} = A_0^{C_{60}} - A_i^{C_{60}} = \Delta A_i^{C_{60}},$$

where $\Delta A_i^{C_{60}}$ expresses how much the intensity of the Raman peak corresponding to the undoped C_{60} has changed. The index i in the above equations denotes the i-th intercalation

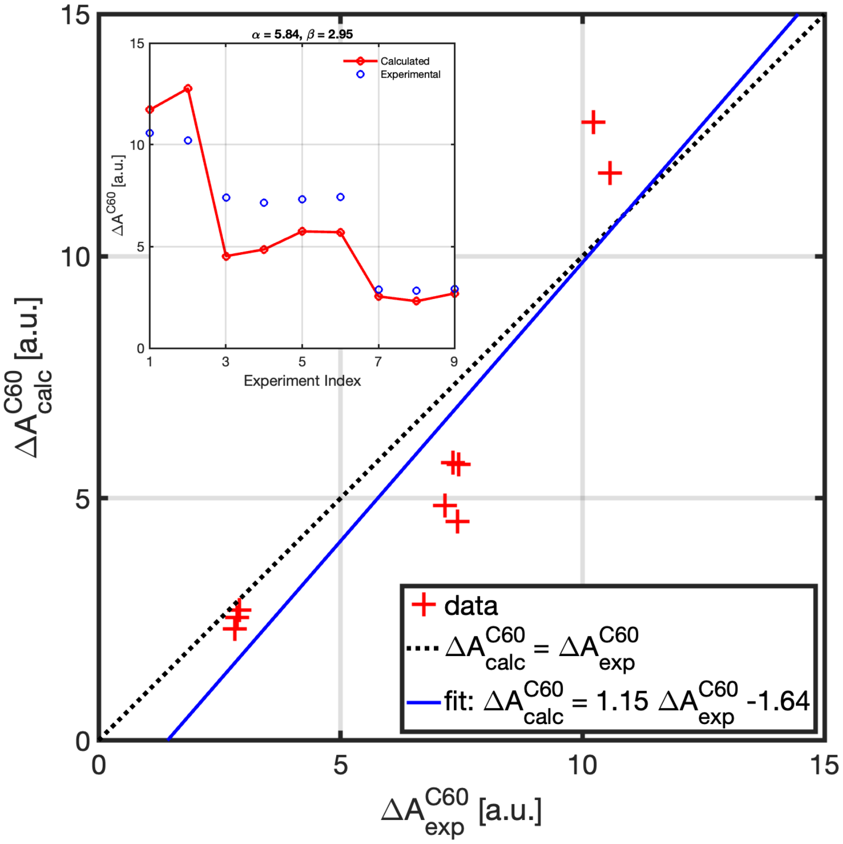

Figure S2: Regression for the fit of Raman cross sections α and β. The experimental versus the calculated Ag(2) peak area is plotted for the fitted values of α and β.

step. In general, we can accurately determine α and β from several sets of Raman intensities $A_i^{C_{60}}$, $A_i^{Rb_1C_{60}}$, and $A_i^{Rb_3C_{60}}$.

All Raman spectra used in this analysis were recorded from the same thin film of C_{60} deposited on a single substrate and sequentially doped with Rb. Each spectrum corresponds to a distinct doping level and is treated as a separate Rb_xC_{60} sample originating from the same initial, undoped C_{60} film with intensity $A_0^{C_{60}}$. The film thickness, laser excitation wavelength (λ =532 nm), accumulation time, and optical alignment were kept constant throughout the measurement series.

By evaluating this relationship for multiple independently measured Rb_xC_{60} samples with

different doping levels x_i, we obtain an overdetermined system of linear equations:

$$\begin{pmatrix} A_1^{\mathrm{Rb_1C_{60}}} & A_1^{\mathrm{Rb_3C_{60}}} \\ A_2^{\mathrm{Rb_1C_{60}}} & A_2^{\mathrm{Rb_3C_{60}}} \\ \vdots & \vdots \\ A_n^{\mathrm{Rb_1C_{60}}} & A_n^{\mathrm{Rb_3C_{60}}} \end{pmatrix} \cdot \begin{pmatrix} \alpha \\ \beta \end{pmatrix} = \begin{pmatrix} \Delta A_1^{\mathrm{C_{60}}} \\ \Delta A_2^{\mathrm{C_{60}}} \\ \vdots \\ \Delta A_n^{\mathrm{C_{60}}} \end{pmatrix},$$

or in short notation

$$\left(\widehat{A_i}\right)_{n\times 2} \cdot \begin{pmatrix} \alpha \\ \beta \end{pmatrix} = \left(\Delta A_i^{\mathrm{C_{60}}}\right)_{n\times 1}.$$

Experimentally, we have used more than two datapoints and hence the number of equations exceeds the number of unknowns. Thus, we solve this system using the method of least squares. The least-squares solution minimizes the squared residual norm

$$R = \left| \left(\widehat{A_i}\right) \cdot \begin{pmatrix} \alpha \\ \beta \end{pmatrix} - \left(\Delta A_i^{\mathrm{C_{60}}}\right) \right|^2 .$$

We perform a least-squares fit that minimizes R, the deviation between experimental points and the model in equation 1 and yields the optimal values of α and β. If we consider a Rb intercalation series with increasing Rb deposition, we can use the identical initial intensity $A_0^{\mathrm{C_{60}}}$ for all points. If several starting samples are used, we generally have a different $A_0^{\mathrm{C_{60}}}$ for each sample. From the regression analysis shown in Figure S2 we obtain $\alpha = 5.84$ and $\beta = 2.95$. The stoichiometry of each $\mathrm{Rb}_x\mathrm{C_{60}}$ sample can be calculated as

$$x_i = \alpha \cdot \frac{A_i^{\mathrm{Rb_1C_{60}}}}{A_0^{\mathrm{C_{60}}}} + 3\beta \cdot \frac{A_i^{\mathrm{Rb_3C_{60}}}}{A_0^{\mathrm{C_{60}}}}.$$

Here we use the fact that $\mathrm{Rb_1C_{60}}$ has one Rb atom per $\mathrm{C_{60}}$ and $\mathrm{Rb_3C_{60}}$ has three Rb atoms per $\mathrm{C_{60}}$ and that they have Raman cross sections α and β, respectively.

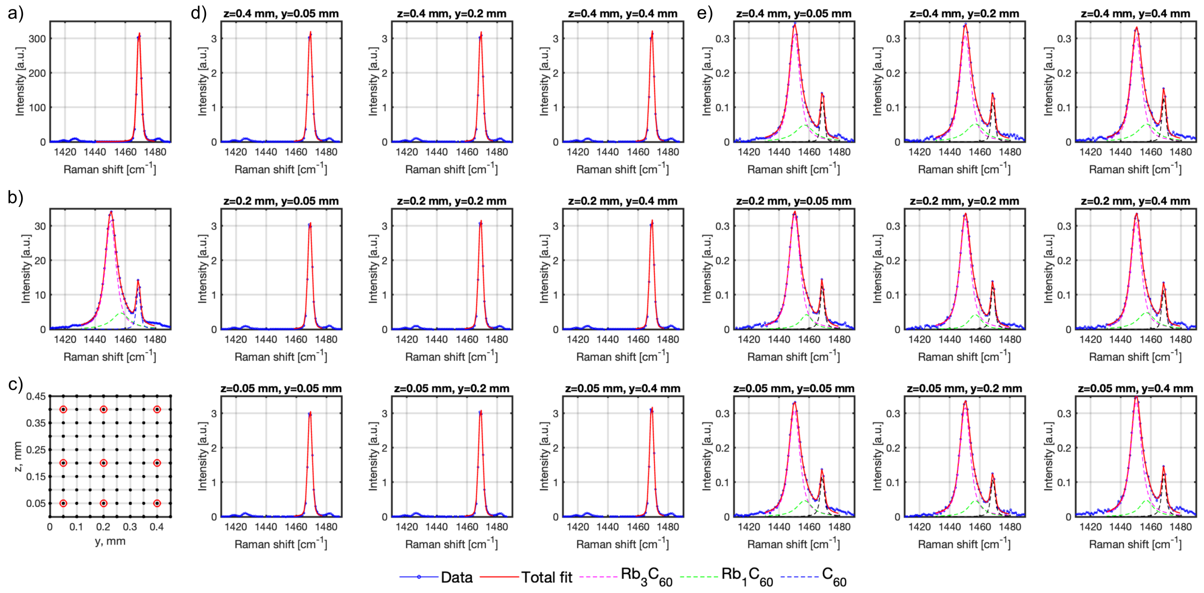

Figure S3: Integrated Raman spectrum of (a) C_{60} immediately after C_{60} deposition and (b) of Rb doped C_{60} immediately after Rb deposition. (c) denotes the location where the map was taken. (d) and (e) denote individual scans of the Raman spectra for C_{60} and Rb-doped C_{60} shown in Figure 2 of the main manuscript.

Raman mapping

Figure S3 depicts Raman spectra at different doping levels from the Raman map shown in Figure 2 of the main paper.

Raman line shape analysis

Figure S4 shows the fits that have been used to determine the stoichiometry x of Rb_xC_{60} in Figure 2f of the main article. The line shape analysis contains peaks of pristine C_{60}, Rb_1C_{60} and Rb_3C_{60}.

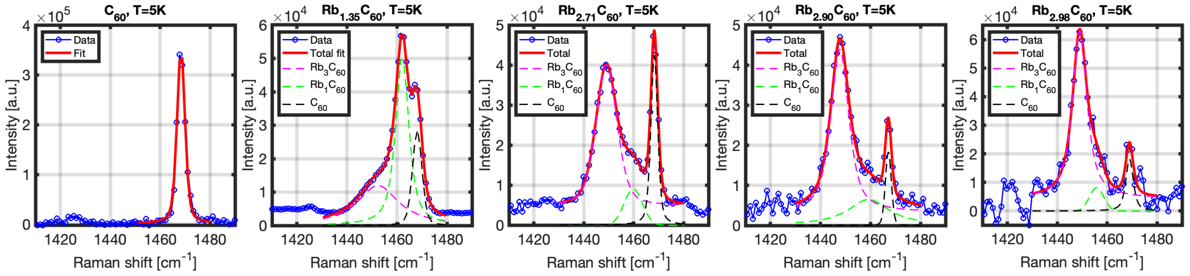

Figure S4: Fits of the Raman spectra of Rb_xC_{60} shown in Figure 2f using components corresponding to pure C_{60}, Rb_1C_{60} and Rb_3C_{60}.

Rb_3C_{60} film thickness dependence of the superconducting transition

Figure S5 depicts the Raman spectra and the temperature dependence of the resistivity $\rho(T)$ for two films with thicknesses of 12 nm and 30 nm. The Raman intensity of the spectra shown in the left panel confirms the film thicknesses (the 30 nm film has stronger intensity than the 12 nm film). The resistivity in the right panel shows that the normal-state $\rho(T)$ and superconducting response have a thickness dependence, consistent with bulk transport through the intercalated Rb_3C_{60} film.

Resistance of the Rb network

We estimate the resistance of the residual Rb network in between the grains. To that end we model the residual resistance via a Rb wire network as shown in Figure S6(a) using the lattice Green's function method[1]. The Rb wires have circular cross section with a diameter $d = 0.6$ nm and a length $a = 50$ nm. The resistance is calculated using realistic parameters as a function of n, the number of channels which we varied in our calculation. Here we used a Rb resistivity $\rho = 1.28 \times 10^{-7}$ Ωm and simulated the resistance for an injected current to 10 μA. The simulated resistance is shown in Figure S6(b). The calculated resistance converges to a value of 2.5 kΩ. This is much higher than the resistance of Rb_3C_{60} and we

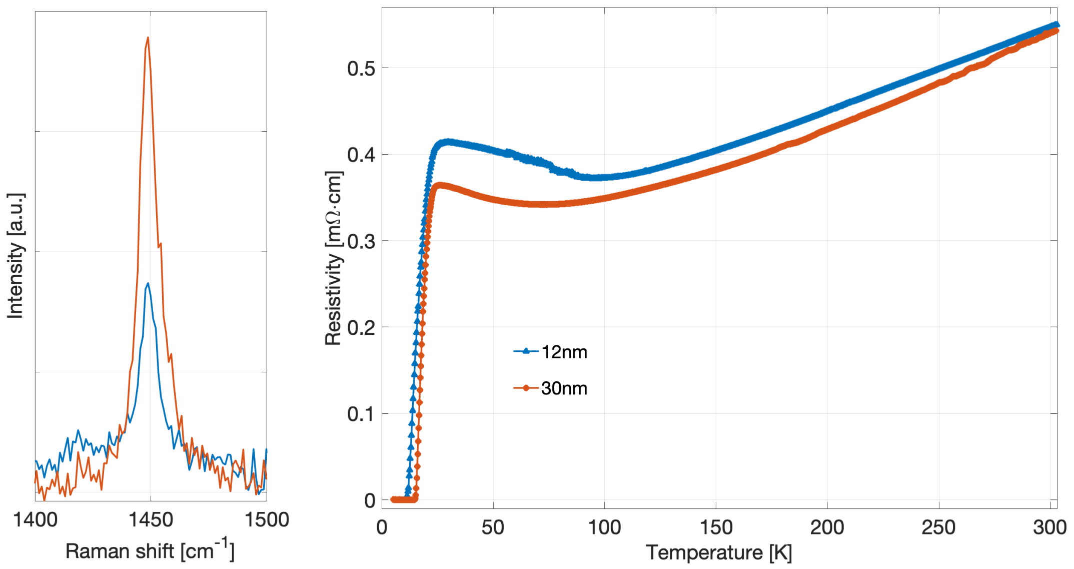

Figure S5: Left panel: Normalised Raman intensity of the Ag(2) mode of Rb3C60 films with 12 nm and 30 nm thickness (blue and red, respectively). Right panel: Temperature-dependent resistivity $\rho(T)$ of Rb_3C_{60} for the two films shown in the left panel.

thus assume that in the effective parallel circuit of Rb_3C_{60} and residual Rb resistance, the Rb_3C_{60} resistance dominates.

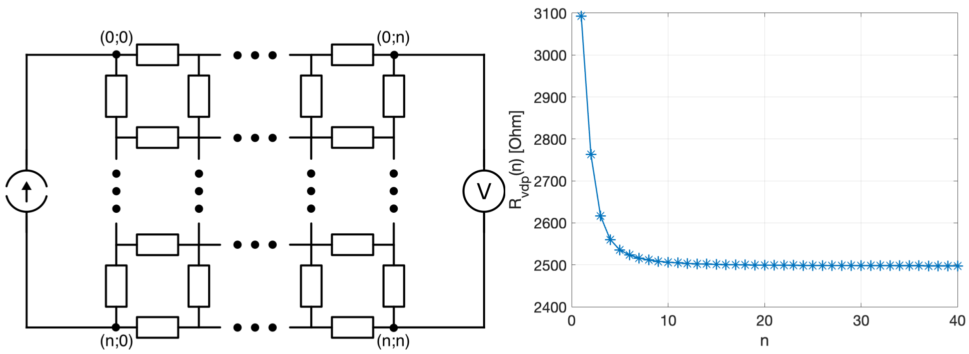

Figure S6: (a) resistance network with n nodes. (b) calculated total resistance of the resistance network in (a) as a function of n.

Stoichiometry determination for $\rho(T)$ measurements

Figure S7 shows the lineshape analysis for the fits that have been used to determine the stoichiometry x of Rb_xC_{60} in Figure 2g of the main paper and the $\rho(T)$ for each sample.

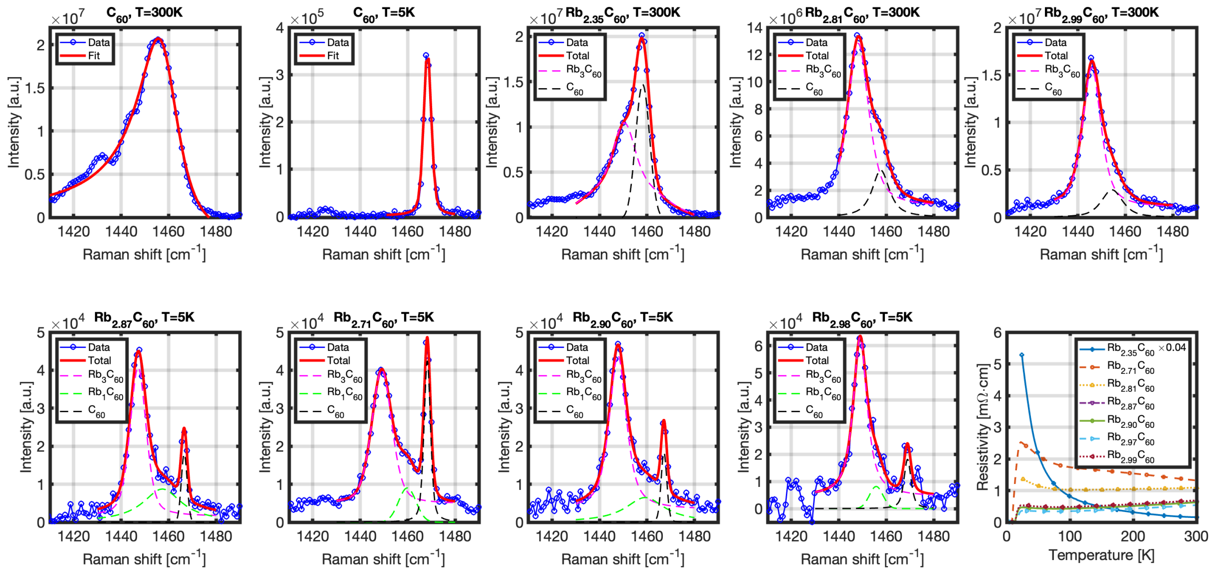

Figure S7: Stoichiometry determination and $\rho(T)$ measurements for each stoichiometry (see Figure 2g of the main paper).

Ionic current

We estimate what fraction of the 10 μA current is ionic. The ionic current required to explain the progression of stoichiometry from $x = 2.5$ at $t = 5.22\times10^5$ s to $x = 2.69$ at $t = 7.97\times10^5$ s (Figures 3d and f from the main paper) yielding $\Delta x \approx 0.2$ in $\Delta t \approx 2.5 \times 10^5$ s. The system geometry is a layer of thickness $w \approx 15$ nm and sides with $L \approx 5$ mm. The density of C_{60} molecules is $\rho \approx 1.3 \times 10^{27}$ /m^3, the volume of the film is $V = wL^2 \approx 4 \times 10^{-13}$ m^3 and the number of C_{60} molecules is $N_{C60} \sim 5 \times 10^{14}$. The required ionic current to explain the observed stoichiometry progression is $I_{Rb+} = e\frac{\Delta x}{\Delta t}N_{C60} \approx 60$ pA. This is 5 orders of magnitude lower than the current of 10 μA which was used in the experiment. We thus conclude that the current is mainly electronic.

Processes I: vdW intercalation

We examine how Rb atoms can traverse the interfacial region between the Rb and C_{60}. Process I explains why a fraction of deposited Rb intercalates immediately into C_{60} after Rb deposition. For calculation of the barriers we constructed model structures consisting of C_{60} and Rb with mixed or uniform gap sizes in between them as shown in Figure S8. In the vdW-10 Å model shown in Figure S8(a-c), the initial configuration contains two Rb-C_{60} interfaces: one being separated only by a vdW gap and the other by a 10 Å vacuum gap. Upon structural relaxation, the Rb atoms migrate to close the vdW gap, whereas the larger 10 Å gap remains open. A similar behavior is observed in the vdW-20 Å models (Figure S8d-f). Because relaxation occurs without an activation barrier, the Rb migration across a vdW gap is effectively barrierless. In contrast to the vdW case, the migration of Rb across the larger gaps requires activation. For the 10 Å-10 Å model, the climbing image-nudged elastic band method (CI-NEB) calculation yields a migration barrier of 0.24 eV (Figure S8c, top), and for the 20 Å-20 Å model the barrier increases to 0.92 eV (Figure S8f, top). Bader charge analysis along the NEB pathway (Figures S8c and f, bottom) show that the migrating Rb atom is neutral on the left side of the transition state (TS) where the driving force arises mainly from the cohesive energy of the Rb slab. Past the TS, the Rb atom becomes positively charged and is driven forward by vdW and Coulombic attractions to the negatively charged C_{60}. Summarizing process I, the migration barrier increases with the interfacial gap size in our static models. This barrier highlights why electric polarization is required for continued Rb migration across the interfacial gaps.

Process II: Diffusion

Process II describes diffusion of Rb^{+} inside the C_{60} grain. We consider two limiting cases: first a Rb-poor case where one Rb^{+} is intercalated, simulating single-ion migration in an otherwise empty lattice. Second, we consider a Rb-rich case where a single Rb vacancy is

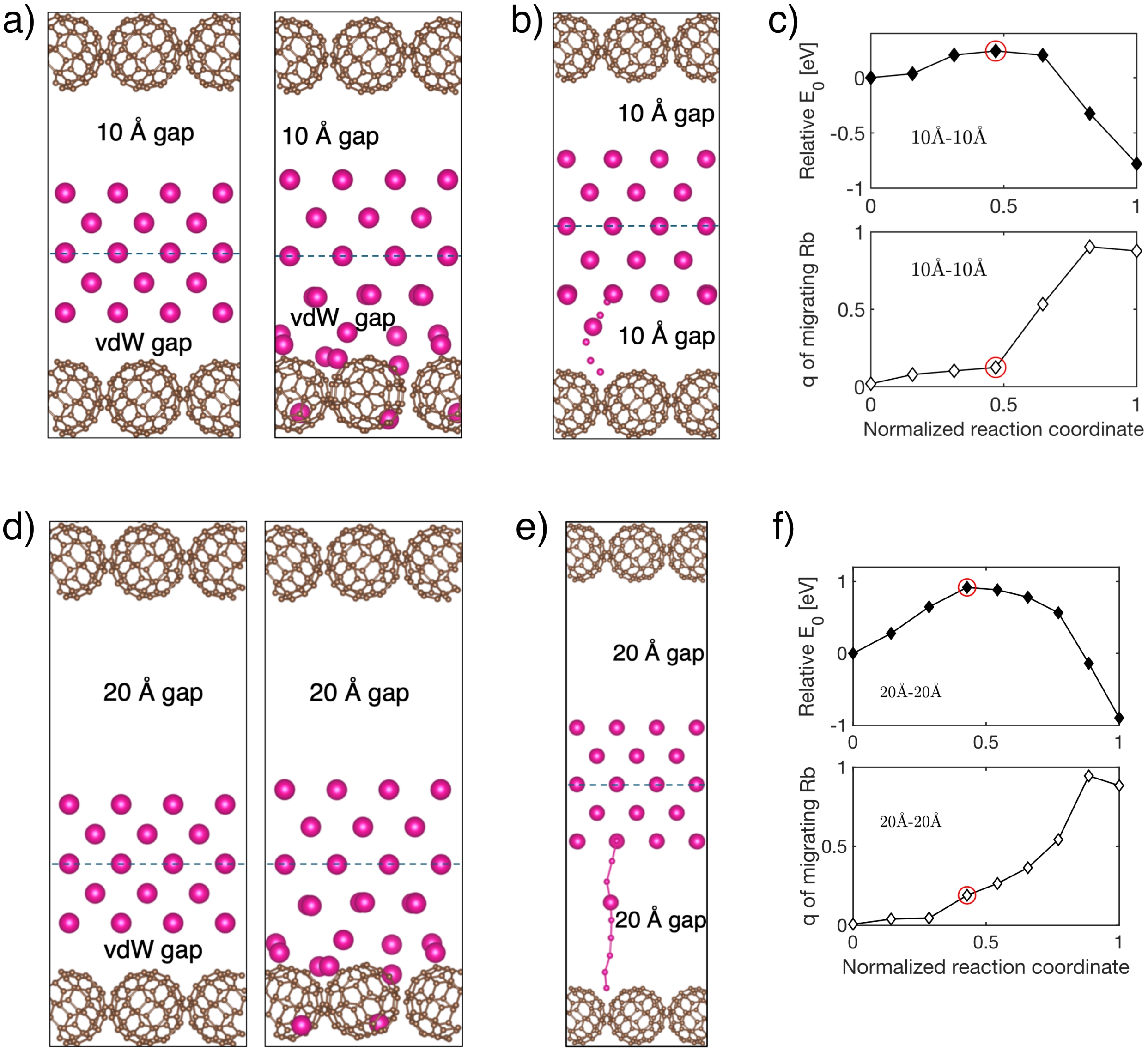

Figure S8: (a) Rb migration across interfacial gaps using a vdW-10 Å model before (left) and after (right) relaxation. (b) CI-NEB pathway for Rb migration across a 10 Å gap in a 10 Å-10 Å model, where the small spheres trace the migration path and the larger sphere denotes the transition state (TS). (c) CI-NEB energy profile and Bader charge (in unit charges e) of migrating Rb atom. The TS is marked by the red circle. (d-f) are equivalent to (a-c) but for migration across a 20 Å gap instead of a 10 Å gap.

introduced, mimicking hole/vacancy migration in a fully intercalated Rb_3C_{60} lattice. The $Fm\bar{3}m$ structure offers two types of interstitial voids for Rb occupation: tetrahedral centers of T_d point group symmetry (denoted as T_d) and octahedral sites of O_h point group symmetry (denoted as O_h), with eight T_d and four O_h voids per unit cell. Through visual inspection of the crystal geometry, we identified three main symmetry-equivalent migration channels: (1) $T_d \rightarrow T_d$ hops along the (100) direction, e.g., from $(1/4, 1/4, 1/4)$ to $(3/4, 1/4, 1/4)$, (2) $T_d \rightarrow T_d$ and $O_h \rightarrow O_h$ hops along face diagonals (110), e.g., $(1/4, 1/4, 1/4)$ to $(3/4, 3/4, 1/4)$, and $(0, 0, 1/2)$ to $(1/2, 1/2, 1/2)$ and (3) $T_d \rightarrow O_h$ hops along body diagonals (111), e.g., $(1/4, 1/4, 1/4)$ to $(1/2, 1/2, 1/2)$. Channels along the (111) direction appear periodically blocked by surrounding C_{60}, necessitating zig-zag or indirect migration paths. NEB calculations reveal that direct $O_h \rightarrow O_h$ transitions are not viable; instead, the Rb^+ ion migrates through an intermediate T_d site. For example, migration from $(0, 0, 1/2)$ to $(1/2, 1/2, 1/2)$ occurs via an adjacent T_d site such as $(1/4, 1/4, 1/4)$. Similarly, transitions along (100) directions follow $O_h \rightarrow T_d \rightarrow O_h$ pathways. The calculated energy barrier for the $T_d \rightarrow O_h$ hop is ~0.19 eV, with the O_h site lying about 0.05 eV higher in energy than the T_d site. A similar migration profile was observed for hole/vacancy hopping in the Rb-rich limit with the energy barrier of ~0.485 eV. Figure S9 shows the calculated barriers for the Rb-poor and Rb-rich case. We have calculated the barriers using CI-NEB.

Our results highlight that effective Rb transport occurs via a repeating $O_h \rightarrow T_d \rightarrow O_h$ sequence along symmetry-equivalent directions, constrained by the fullerene cage network. Our simulation for Rb rich-limit case (11 Rb atoms and 4 C_{60} units) effectively corresponds to Rb_xC_{60} with $x = 2.5$. Using a standard hopping model for the diffusion constant $D = (a^2\nu/6)\exp(-E_a/k_BT)$ with a jump distance of $a = 0.65$ nm and a calculated average attempt frequency (see next section) of $\nu^{\text{eff}} \approx 8.1 \times 10^{12}$ s^{-1} the Rb-rich limit barrier of $E_a = 0.485$ eV corresponds to a diffusion coefficient $D \sim 4 \times 10^{-15}$ m^2/s at 300 K. The diffusion time is estimated as $t = L^2/(2dD)$ with dimensionality $d = 1$ for thickness limited diffusion and a film thickness $L = 15$ nm as $t \sim 10^{-2}$ s. Hence t is negligible compared to the

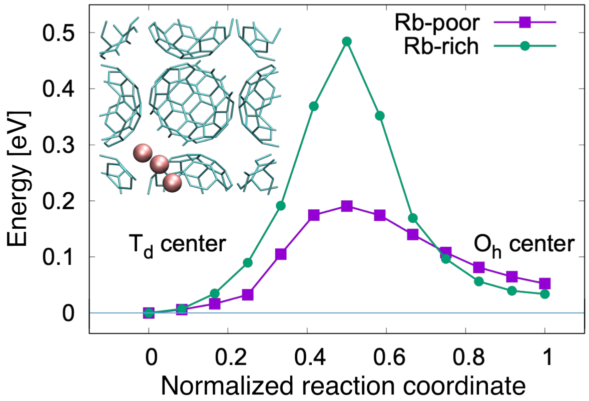

Figure S9: CI-NEB energy profiles for Rb^+ migration within an Rb_xC_{60} grain between T_d and O_h vacancy sites. The Rb-poor (Rb-rich) case corresponds to a single Rb atom (vacancy) per unit cell migrating in an otherwise pristine C_{60} (Rb_3C_{60}) lattice. The inset shows the Rb^+ ion moving along the NEB path between adjacent T_d and O_h sites.

observed time scales for the intercalation. Processes I and II provide fast local equilibration of available Rb^+ within and around individual grains. However, this easily available Rb is generally not sufficient to synthesize Rb_3C_{60}. As we will show in the following, an applied bias is required to reach Rb_3C_{60}.

Estimate of the Rb^+ diffusion coefficient and intercalation time

To obtain a more realistic estimate of the Rb^+ diffusion coefficient in Rb_3C_{60}, we complemented our DFT CI-NEB barriers with attempt frequencies extracted from finite-temperature molecular dynamics using Grimme's extended tight-binding (xTB) model[2,3] as implemented in the DFTB+ code[4,5]. In contrast to the commonly used ad hoc prefactor $\nu \sim 10^{13}\,\mathrm{s}^{-1}$, our estimate of ν is based on the local vibrational dynamics of Rb^+ in the Rb-rich lattice.

We first optimized the Rb_3C_{60} unit cell using the xTB Hamiltonian in DFTB+ with Grimme's parametrization, and used the resulting lattice constant $a_0 = 14.425$ Å in all subsequent xTB-based molecular dynamics. The Rb_3C_{60} structure is modeled in the $Fm\bar{3}m$ phase, with four C_{60} molecules and twelve Rb atoms per primitive cell. Eight Rb occupy tetrahedral (T_d) voids and four Rb occupy octahedral (O_h) voids.

Finite-temperature dynamics were carried out with the xTB model in DFTB+ in the

NVT ensemble at $T = 300$ K, using a Nosé–Hoover thermostat, a time step of 1 fs, and a total simulation length of 5 ps. During these runs we monitored selected Rb atoms that remained localized in either an O_h or T_d well (i.e., without actual hopping events), so that the trajectories reflect small-amplitude oscillations around the minima rather than activated barrier crossings.

To define the geometric hop between neighboring Rb sites, we identified one representative pair of adjacent voids in fractional coordinates: a T_d site at $(\frac{1}{4}, \frac{1}{4}, \frac{1}{4})$ and an O_h site at $(\frac{1}{2}, 0, 0)$. The corresponding fractional displacement is

$$\Delta\mathbf{f} = \mathbf{f}_{\mathrm{Td}} - \mathbf{f}_{\mathrm{Oh}} = \left(-\tfrac{1}{4}, \tfrac{1}{4}, \tfrac{1}{4}\right),$$

which, when multiplied by the xTB-optimized lattice constant a_0, gives the Cartesian jump vector

$$\Delta\mathbf{r} = a_0\,\Delta\mathbf{f} = a_0\left(-\tfrac{1}{4}, \tfrac{1}{4}, \tfrac{1}{4}\right).$$

The corresponding hop distance entering the diffusion expression is

$$a = \|\Delta\mathbf{r}\| = a_0\,\frac{\sqrt{3}}{4} \approx 6.5\ \text{Å},$$

which we take as the characteristic separation between neighboring Rb residence centers.

For the purpose of extracting attempt frequencies, we approximated the reaction coordinate by the straight line connecting the two minima. That is, for the Rb atom of interest we constructed a unit vector

$$\hat{\mathbf{e}} = \frac{\mathbf{R}^{\mathrm{Rb}}_{\mathrm{Td}} - \mathbf{R}^{\mathrm{Rb}}_{\mathrm{Oh}}}{\left\|\mathbf{R}^{\mathrm{Rb}}_{\mathrm{Td}} - \mathbf{R}^{\mathrm{Rb}}_{\mathrm{Oh}}\right\|},$$

where $\mathbf{R}^{\mathrm{Rb}}_{\mathrm{Oh}}$ and $\mathbf{R}^{\mathrm{Rb}}_{\mathrm{Td}}$ denote the Cartesian coordinates of Rb in the O_h and T_d minima, respectively. During the NVT trajectory, we recorded the Rb velocity vector $\mathbf{v}(t)$ at each

time step and projected it onto this direction,

$$v_{\parallel}(t) = \mathbf{v}(t) \cdot \hat{\mathbf{e}},$$

which yields a one-dimensional velocity time series along the approximate hop direction.

From $v_{\parallel}(t)$ we computed the velocity autocorrelation function (VACF),

$$C_{vv}(t) = \langle v_{\parallel}(0)\, v_{\parallel}(t) \rangle,$$

where the average is taken over time origins along the trajectory. The Fourier transform of $C_{vv}(t)$ gives the vibrational power spectrum along the hop direction. For Rb in an O_h site we obtained a dominant low-frequency peak at $\tilde{\omega}_{\mathrm{Oh}} \approx 40\ \mathrm{cm}^{-1}$, while for Rb in a T_d site the corresponding peak is at $\tilde{\omega}_{\mathrm{Td}} \approx 55\ \mathrm{cm}^{-1}$ which yields the local attempt frequencies for small-amplitude oscillations of Rb^+ along a single escape direction out of an O_h or T_d well

$$\nu_{\mathrm{Oh}} \approx 1.2 \times 10^{12}\ \mathrm{s}^{-1}, \qquad \nu_{\mathrm{Td}} \approx 1.65 \times 10^{12}\ \mathrm{s}^{-1}.$$

In the $Fm\overline{3}m$ Rb_3C_{60} lattice, each O_h site has eight symmetry-equivalent neighboring T_d sites, whereas each T_d site is connected to four equivalent O_h neighbors. We therefore define effective attempt frequencies

$$\nu_{\mathrm{Oh}}^{\mathrm{eff}} = z_{\mathrm{Oh}}\, \nu_{\mathrm{Oh}}, \qquad \nu_{\mathrm{Td}}^{\mathrm{eff}} = z_{\mathrm{Td}}\, \nu_{\mathrm{Td}},$$

with coordination numbers $z_{\mathrm{Oh}} = 8$ and $z_{\mathrm{Td}} = 4$, leading to

$$\nu_{\mathrm{Oh}}^{\mathrm{eff}} \approx 9.6 \times 10^{12}\ \mathrm{s}^{-1}, \qquad \nu_{\mathrm{Td}}^{\mathrm{eff}} \approx 6.6 \times 10^{12}\ \mathrm{s}^{-1}.$$

To estimate the Rb^+ diffusion coefficient, we combine these xTB-based attempt frequencies with the migration barriers obtained from CI-NEB calculations at the DFT level. For

a thermally activated hopping process with hop distance a and activation energy E_a, the diffusion constant in three dimensions is approximated by

$$D = \frac{a^2}{6}\,\nu^{\text{eff}}\,\exp\!\left(-\frac{E_a}{k_{\text{B}}T}\right),$$

where ν^{eff} is the effective attempt frequency for the relevant site type and k_{B} is Boltzmann's constant. For the Rb-rich (vacancy) migration pathway, we use the DFT barrier $E_a = 0.485$ eV and ν^{eff} in the range defined by $\nu^{\text{eff}}_{\text{Td}}$ and $\nu^{\text{eff}}_{\text{Oh}}$ above. At $T = 300$ K this yields diffusion coefficients on the order of

$$D \sim (3\text{–}5) \times 10^{-15}\ \text{m}^2\,\text{s}^{-1},$$

which are only slightly smaller than the estimate based on a generic $\nu = 10^{13}\ \text{s}^{-1}$. Using this D, the characteristic time to diffuse across a distance L can be estimated as

$$t_{\text{diff}} \sim \frac{L^2}{2d\,D},$$

where d is the dimensionality ($d = 1$ for purely thickness-limited diffusion, $d = 3$ for isotropic diffusion). For a 15 nm C_{60} film thickness, $L \approx 15$ nm and $D \sim 10^{-15}$–$10^{-14}\ \text{m}^2\,\text{s}^{-1}$ give characteristic times on the order of

$$t_{\text{diff}} \sim 10^{-2}\ \text{s}.$$

Thus, even with the more conservative xTB-based prefactor, Rb^+ diffusion within the Rb_3C_{60} phase is extremely fast on experimental timescales. This supports the conclusion that internal diffusion (Process II) is not rate-limiting, and that the multi-day electro-intercalation kinetics are instead controlled by the interfacial charge-transfer step (Process III) rather than by slow Rb^+ motion inside the fulleride grains.

Process III: Details of the Butler-Volmer model

We employ the Butler-Volmer equation for interfacial ionic current density $j(\eta) = e\Gamma_s[k_{\rm f}(\eta) - k_{\rm b}(\eta)]$, where $k_{\rm f}, k_{\rm b}$ are forward/backward rate constants and Γ_s is the effective number of sites on the C_{60} surface. Using transition-state theory (TST) with transmission factor $\kappa \approx 1$ and a small overpotential η we get an interfacial ionic current density per unit surface of Rb|C_{60} interface:

$$j(\eta) \approx \frac{\kappa \Gamma_s e^2 \eta}{2\pi\hbar} \exp\Big(-\frac{\Delta G_0^\ddagger}{k_B T}\Big) \exp(-2\kappa_{\rm tun} d),$$

where $\Delta G^\ddagger$ is the free energy barrier and the last term is a tunneling rate of the electron throughout the gap of length d.

We denote $\ell = 50$ nm the typical grain size of C_{60} and ΔV the total voltage drop. Thus, we estimate $\eta = (\ell/L)\Delta V \ll k_B T/e$. The total ionic current is the surface current density times the effective surface, which we take as a section of the device for each layer of grains, that is $wL \times L/\ell$. Thus,

$$I_{\rm Rb^+} \approx \frac{\kappa w L \Gamma_s e^2 \Delta V}{2\pi\hbar} \exp\Big(-\frac{\Delta G_0^\ddagger}{k_B T}\Big) \exp(-2\kappa_{\rm tun} d).$$

Closer to $x \to 3$, we model saturation with a phenomenological Avrami model. Using a current $I_{\rm Rb^+}$ which does not depend on the grain size, the stoichoimetry dynamics is given by:

$$x(t) = 3\left[1 - \exp\left(-\frac{I_{\rm Rb^+}}{3eN_{\rm C_{60}}} t\right)\right]$$

from which we can extract an effective ionic current:

$$I^{\rm eff}_{\rm Rb^+}(t) = e\frac{dx(t)}{dt} N_{\rm C_{60}} = I_{\rm Rb^+} \cdot \exp\left(-\frac{I_{\rm Rb^+}}{3eN_{\rm C_{60}}} t\right).$$

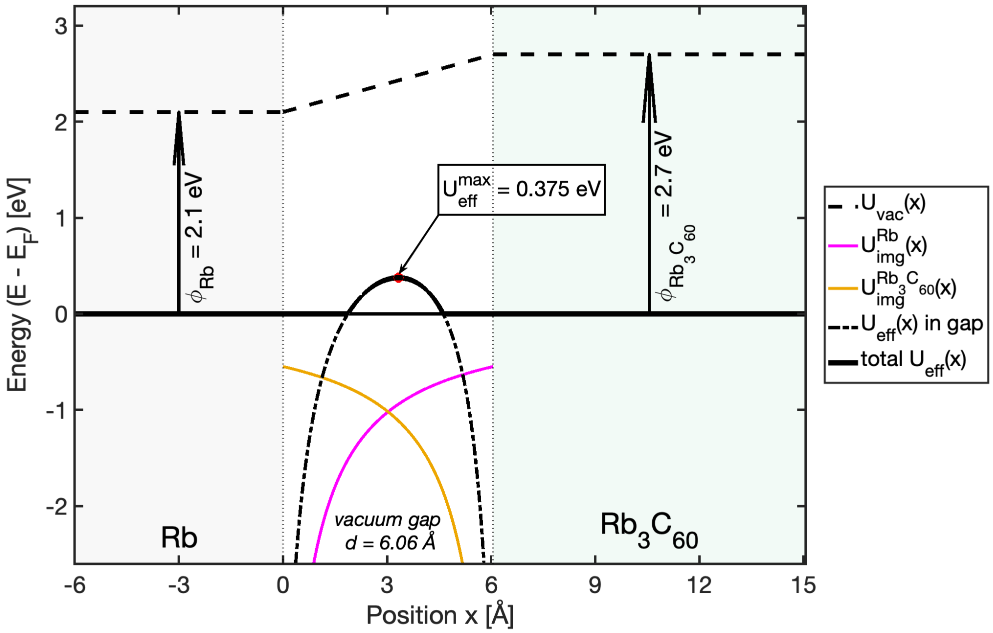

Figure S10: Energy diagram of the vacuum tunneling barrier between metallic Rb and the metallic Rb_3C_{60} shell.

Tunnel barrier between Rb and Rb_3C_{60}

Vacuum tunneling of the electron takes place between the Rb metal and the Rb_3C_{60} shell. The small gap in between Rb and Rb_3C_{60} is a tunneling barrier with the height set by the work functions (2.1 eV and 2.7 eV) (Figure S10). The width of that barrier is larger than the van-der-Waals distance (4.7 Å). When an electron tunnels from Rb to Rb_3C_{60}, an image charge in both metals will be induced which lowers the effective barrier. In addition, an applied field modulates the effective barrier. Here we estimate the tunneling barrier by adding together all contributions. The effective potential barrier between the metallic Rb_3C_{60} grain and the surrounding Rb shell is taken as $U_{\text{eff}}(x) = U_{\text{vac}}(x) + U_{\text{img}}(x)$, where $U_{\text{vac}}(x) = \phi_{\text{Rb}} + \frac{\phi_{\text{Rb}_3\text{C}_{60}} - \phi_{\text{Rb}}}{d}\,x$, describes the linear trapezoidal vacuum level across the gap of width d, and $U_{\text{img}}(x) = -\frac{e^2}{16\pi\varepsilon_0}\left[\frac{1}{x+x_0} + \frac{1}{(d-x)+x_0}\right]$ represents image charge lowering near both metallic interfaces. Here x_0 is an effective distance, which is used to remove the singularity at the metal surface and typically in the range of atomic size ($x_0 = 0.5$ Å). For the values used in the manuscript ($\phi_{\text{Rb}} = 2.1$ eV, $\phi_{\text{Rb}_3\text{C}_{60}} = 2.7$ eV, $d = 6.06$ Å), we can estimate the

maximum of the effective potential barrier as $U_{\rm eff}^{max} = 0.375$ eV.

Rb ionization and electromigration

Large electric fields can in principle directly ionize Rb but experimental measurements of field ionisation probabilities for Rb atoms show that electric fields of order $E_{\rm ion} \sim 3 \times 10^9$ V/m[6] are required. This is eight orders of magnitude larger than the fields in our sample and we thus exclude field-induced ionisation of neutral Rb. If a large electronic current is passed through the sample, the resulting electron wind exerts a force onto the ions of the conductor which is known as electromigration. Electromigration was qualitatively described for Aluminum ion transfer in Silicon.[7] In the drift–diffusion framework the electromigration-driven ionic current density follows the Nernst–Planck relation $j_{\rm EM} = \frac{e^2}{k_B T} cDZE$, where c is the Rb concentration, D is the Rb diffusion coefficient, Z is the effective valence and E is the electric field[7,8]. The corresponding total electromigration current is $I_{\rm EM} = j_{\rm EM} A$ with the metallic cross-section $A = wW$ of the Rb leakage path, where $w = 15$ nm is film thickness and $W = 50$ μm is the width of leakage channel. We use experimental parameters $E \approx 44$ V/m (from $\Delta V = I_{\rm total} \cdot R \approx 10$ μA $\cdot 22$ k$\Omega \approx 0.22$ V), temperature $T = 300$ K, $n_{\rm C_{60}} \approx 1.38 \times 10^{27}$ m^{-3}, $x = 2.5$ (so total Rb density $n_{\rm Rb} = x n_{\rm C_{60}} \approx 3.45 \times 10^{27}$ m^{-3}), and realistic mobile fraction $f_{\rm mob} = 1\%$ ($c = f_{\rm mob}\, x n_{\rm C_{60}}$). For a Rb diffusion coefficient $D = 10^{-15} \div 10^{-14}$ m^2/s and effective valence $Z = 1 \div 10$, the resulting electromigration current is $I_{\rm EM} \sim 10^{-7} \div 10^{-5}$ pA. Comparing $I_{\rm EM}$ to the ion current required for intercalation (I_{Rb^+}=60 pA) reveals that $I_{\rm EM}$ electromigration of Rb can be ignored.

Electron-phonon coupling of Rb intercalated fullerene

Figure S11 shows the fit of Raman spectra for C_{60} and Rb_3C_{60} by Lorentzians. The increase in the linewidths have been used to determine λ, the EPC constant.

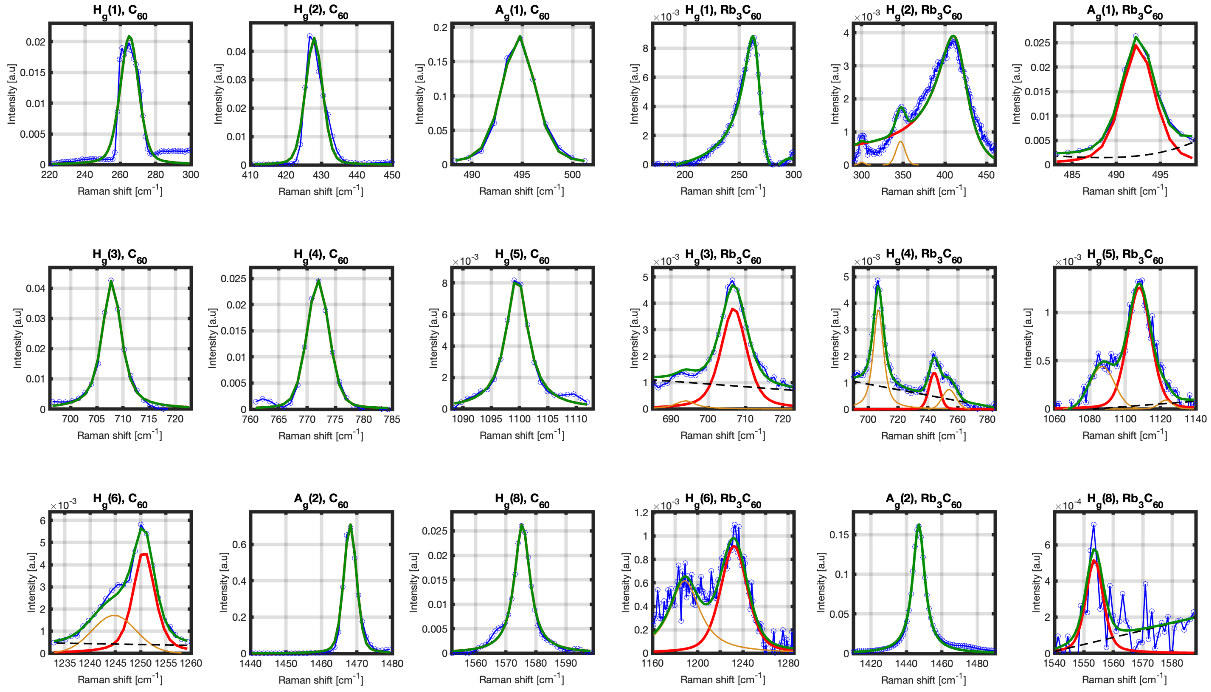

Figure S11: Fits to Raman spectra of C_{60} and Rb_3C_{60}. Blue curves are the measured Raman spectra and the green curves the fit of the total Raman spectra. Red and orange curves denote the individual peaks of the fit and black the background. The FWHM of these fits are plotted in Figure 5 of the main paper.

References

1. Cserti, J. Application of the lattice Green's function for calculating the resistance of an infinite network of resistors. Am. J. Phys. **2000**, 68, 896–906.

2. Grimme, S.; Antony, J.; Ehrlich, S.; Krieg, H. A consistent and accurate ab initio parametrization of density functional dispersion correction (DFT-D) for the 94 elements H-Pu. J. Chem. Phys. **2010**, 132, 154104.

3. Grimme, S.; Ehrlich, S.; Goerigk, L. Effect of the damping function in dispersion corrected density functional theory. J. Comput. Chem. **2011**, 32, 1456–1465.

4. Hourahine, B.; Berdakin, M.; Bich, J. A.; Bonafe, F. P.; Camacho, C.; Cui, Q.; Deshaye, M. Y.; Diaz Miron, G.; Ehlert, S.; Elstner, M.; Frauenheim, T.; Goldman, N.; Gonzalez Leon, R. A.; van der Heide, T.; Irle, S.; Kowalczyk, T.; Kubar, T.; Lee, I. S.; Lien-Medrano, C. R.; Maryewski, A. et al. Recent Developments in DFTB+, a Software Package for Efficient Atomistic Quantum Mechanical Simulations. The Journal of Physical Chemistry A **2025**, 129, 5373–5390, PMID: 40479742.

5. Hourahine, B.; Aradi, B.; Blum, V.; Bonafe, F.; Buccheri, A.; Camacho, C.; Cevallos, C.; Deshaye, M. Y.; Dumitrica, T.; Dominguez, A.; Ehlert, S.; Elstner, M.; van der Heide, T.; Hermann, J.; Irle, S.; Kranz, J. J.; Köhler, C.; Kowalczyk, T.; Kubar, T.; Lee, I. S. et al. DFTB+, a software package for efficient approximate density functional theory based atomistic simulations. The Journal of Chemical Physics **2020**, 152, 124101.

6. Wessels, P.; Ruff, B.; Kroker, T.; Kazansky, A. K.; Kabachnik, N. M.; Sengstock, K.; Drescher, M.; Simonet, J. Absolute strong-field ionization probabilities of ultracold rubidium atoms. Communications Physics **2018**, 1, 32.

7. Black, J. R. Electromigration—A brief survey and some recent results. IEEE Transactions on Electron Devices **2005**, 16, 338–347.

8. Lloyd, J. Electromigration in thin film conductors. Semiconductor science and technology **1997**, 12, 1177.
